# Rare and potentially pathogenic variants in hydroxycarboxylic acid receptor genes identified in breast cancer cases

**DOI:** 10.1186/s12920-021-01126-3

**Published:** 2021-12-01

**Authors:** Cierla McGuire Sams, Kasey Shepp, Jada Pugh, Madison R. Bishop, Nancy D. Merner

**Affiliations:** Department of Pathobiology, College of Veterinary Medicine, Auburn University, 165 Greene Hall, Auburn, AL 36849 USA

**Keywords:** Breast cancer, Hydroxycarboxylic acid receptor, G-protein coupled receptor, Genetic variants, And protein elongation

## Abstract

**Background:**

Three genes clustered together on chromosome 12 comprise a group of hydroxycarboxylic acid receptors (HCARs): *HCAR1*, *HCAR2*, and *HCAR3*. These paralogous genes encode different G-protein coupled receptors responsible for detecting glycolytic metabolites and controlling fatty acid oxidation. Though better known for regulating lipid metabolism in adipocytes, more recently, HCARs have been functionally associated with breast cancer proliferation/survival; *HCAR2* has been described as a tumor suppressor and *HCAR1* and *HCAR3* as oncogenes. Thus, we sought to identify germline variants in *HCAR1*, *HCAR2,* and *HCAR3* that could potentially be associated with breast cancer risk.

**Methods:**

Two different cohorts of breast cancer cases were investigated, the Alabama Hereditary Cancer Cohort and The Cancer Genome Atlas, which were analyzed through nested PCRs/Sanger sequencing and whole-exome sequencing, respectively. All datasets were screened for rare, non-synonymous coding variants.

**Results:**

Variants were identified in both breast cancer cohorts, some of which appeared to be associated with breast cancer BC risk, including *HCAR1* c.58C > G (p.P20A), *HCAR2* c.424C > T (p.R142W), *HCAR2* c.517_518delinsAC (p.G173T), *HCAR2* c.1036A > G (p.M346V), *HCAR2* c.1086_1090del (p.P363Nfs*26), *HCAR3* c.560G > A (p.R187Q), and *HCAR3* c.1117delC (p.Q373Kfs*82). Additionally, *HCAR2* c.515C > T (p.S172L), a previously identified loss-of-function variant, was identified.

**Conclusions:**

Due to the important role of HCARs in breast cancer, it is vital to understand how these genetic variants play a role in breast cancer risk and proliferation and their consequences on treatment strategies. Additional studies will be needed to validate these findings. Nevertheless, the identification of these potentially pathogenic variants supports the need to investigate their functional consequences.

**Supplementary Information:**

The online version contains supplementary material available at 10.1186/s12920-021-01126-3.

## Background

There are three known genes clustered together on chromosome 12 that comprise a group of hydroxycarboxylic acid receptors (HCARs), *HCAR1*, *HCAR2*, and *HCAR3*. With extreme homology, these paralogous genes encode three different heterotrimeric G-protein coupled receptors (GPCRs), commonly referred to as GPR81, GPR109A, and GPR109B, respectively. These genes are responsible for detecting glycolytic metabolites and controlling the rate of fatty acid oxidation [1, 2]. GPCRs are characterized by 7-transmembrane domains, an N-terminus that interacts with extracellular components, and a C-terminus responsible for transmitting intracellular signals. Since GPCRs are embedded in the cellular membrane and initiate signal transduction, they eliminate the challenge of intracellular drug administration; thus, they are very effective treatment options targeted by 30–50% of existing pharmaceuticals [3].

Though better known for their regulation of lipid metabolism in adipocytes, more recently, HCARs have been functionally associated with breast cancer (BC) [1, 2, 4–7]. For instance, *HCAR2* is described as a tumor suppressor gene because it exhibits a 70% reduction of cell-surface expression in primary BC cells; and its cellular expression is essential for the initiation of apoptosis by its endogenous ligands [4]. In contrast, *HCAR1* and *HCAR3* are considered oncogenes that show notably increased mRNA expressions in BC cells compared to controls and result in BC cell death when knocked down [5, 6]. Knock-down of *HCAR3* has been demonstrated to result in BC cell death through uncontrolled up-regulation of fatty acid oxidation, which can be mitigated by introducing fatty acid oxidation inhibitors [5]. Furthermore, upon activation by lactate or butyrate, *HCAR1* increases the expression of DNA repair proteins and subsequently increases Hela cells' resistance to doxorubicin [7]. Doxorubicin is a common BC chemotherapy drug. Reduced doxorubicin sensitivity is observed during BC treatment, potentially mediated by extracellular matrix proteins [8]; thus, *HCAR1* could be contributing to the resistance.

The metabolic function, differential expression, and promising therapeutic potential of HCARs make their involvement in BC tumorigenesis an exciting research topic. With potential implications in cancer precision medicine, it is essential to identify inherited genetic variants associated with BC risk, molecular subtype, and drug metabolism. This manuscript details the genetic screening of *HCAR1*, *HCAR2*, and *HCAR*3 in 46 BC cases from the Alabama Hereditary Cancer Cohort (AHCC) and 649 BC cases from The Cancer Genome Atlas (TCGA) [9] to identify rare, inherited variants with potentially damaging effects.

## Methods

### Alabama Hereditary Cancer Cohort (AHCC)

Forty-six unrelated BC cases from the AHCC, who self-described as being White/European American, enrolled in a hereditary cancer genetics research study through Auburn University Institutional Review Board-approved protocols, 14–232 or 15–111, which was previously described by Bishop et al. [9]. Informed consent was obtained through writing for all study participants. In brief, the study criteria included BC-affected individuals with a family history of the disease or diagnosed with BC under 45 years. Blood samples were obtained from all participants, and genomic DNA was extracted for genetic analyses.

### AHCC genetic analyses

Due to the extreme homology between *HCAR1* (NM_032554.3), *HCAR2* (NM_177551.3), and *HCAR3* (NM_006018.2), primers were carefully designed using Primer3Plus to amplify each gene separately through nested PCRs (Additional file 1: Tables S1, S2, and S3 and Figures S1, S2, and S3). External primers were designed for each gene that initially amplified a large (> 3.1 Kb) PCR product from genomic DNA. The forward and reverse external primers were located upstream and downstream of each transcript and uniquely hit once in the genome according to the University of California, Santa Cruz (UCSC) BLAT [10, 11]. Overall, this external primer design increased specificity and avoided amplification of the other paralogous genes. Internal primers were designed to amplify smaller regions of each large amplicon through nested PCRs. For this study, which screened for coding variants in each gene, only internal primers that targeted coding regions were used for mutation analysis (Additional file 1: Tables S1, S2, and S3 and Figures S1, S2, and S3). Touchdown PCRs were carried out using a final annealing temperature of 56 °C. Extension times varied according to amplicon size (1 min/Kb), and betaine was required for GC rich amplicons. Nested PCR amplicons were Sanger sequenced at Eurofins Genomics. Sequences were analyzed using Mutation Surveyor (Soft Genetics). Variant validation followed a similar process as previously described [12], and only rare and non-synonymous coding variants (with European Americans MAFs of < 1% in Exome Variant Server (EVS) [13]) were validated. EVS provides whole-exome sequencing data in aggregate for ~ 4300 individuals, providing ~ 8600 control alleles for assessment.

Some internal primer sets would have amplified multiple products if the template was genomic DNA. To demonstrate that nested PCRs increased specificity, we used UCSC In-silico PCR (hg38 assembly) to determine genomic DNA amplicons [11] and BLASTn to align/compare those amplicons [14, 15]. Electropherograms of our nested PCR products confirmed proper amplification by viewing the specific positions that varied between the predicted/in-silico amplicons, representing *HCAR1, HCAR2,* or *HCAR3* (i.e., Additional file 1: Figure S4, confirming *HCAR3* c.560G > A (p.R187Q)).

In R (v 3.5.1), each validated variant was investigated using Fisher's exact test [16, 17] to generate *p* values comparing allele frequencies between AHCC BC cases and EVS ethnic-matched controls. *p* Values of < 0.05 were considered to be statistically significant and were not corrected for multiple testing. In addition, the pathogenicity of each missense variant was predicted using Polyphen2 [18], and amino acid conservation was assessed using WebLogo [19]. Finally, regarding frameshift variants, Mutalyzer [20], Phyre2 [21], I-TASSER [22, 23], and PSORT [24, 25] were used to determine differences between the wildtype and mutant protein.

### The Cancer Genome Atlas (TCGA)

For the identification of germline variants, a total of 649 blood-derived whole-exome binary sequence alignment mapping (BAM) files were downloaded through the approved research project #10805 using the Genomic Data Commons (GDC) Data Portal Repository. All individuals were categorized as European American or "White" BC cases. For sample acquisition, specific filters in the 'Cases’ category included: Project (TCGA-BRCA), Sample Type (Blood-Derived Normal (NB)), and Race (‘White’). Samples were filtered further in the ‘Files’ category and included Experimental Strategy (WXS) and Data Format (BAM). These files were downloaded using the GDC Data Transfer Tool (version 1.2.0). A total of 650 sample files were obtained for European Americans. Only individuals with known ages of BC onset were included in this study; therefore, one European American BAM file was removed from all subsequent analyses. The 649 BAM files had been previously aligned to the hg38 human reference genome. Upon download, the files were processed using a bioinformatics pipeline adapted from the Genome Analysis Toolkit’s (GATK’s) best practices pipeline. *HCAR1* (NM_032554; chr12:122726076-122730844), *HCAR2* (NM_177551; chr12:122701293-122703357), and *HCAR3* (NM_006018; chr12:122714756-122716811) were extracted from the variant calling format (VCF) files and then annotated using ANNOVAR (version June2017). Variants were filtered to include rare, non-synonymous variants with ethnic-specific minor allele frequencies of < 1% in EVS [13].

Variants (and surrounding nucleotides within the same reads) were visualized using recalibrated BAM files in Integrative Genomic Viewer (IGV) to determine validity and confirm proper alignment, considering the extreme homology between the *HCAR* genes, particularly *HCAR2* and *HCAR3* (Additional file 1: Figures S5, S6, and S7). BLASTn was used to align *HCAR2* and *HCAR3* nucleotide/mRNA sequences to note the sequence differences, aiding in alignment confirmation [14, 15]. For each visually confirmed variant, Fisher’s exact tests were performed to compare allele frequencies in TCGA BC cases versus controls (EVS; European Americans) [13], similar to the above described AHCC individual variant statistical analysis. Additionally, the Fisher method was used for gene-based aggregation analyses for TCGA data; the ‘sumlog’ command was used as part of the ‘metap’ packages in R [26, 27].

## Results

**Fig. 1:**
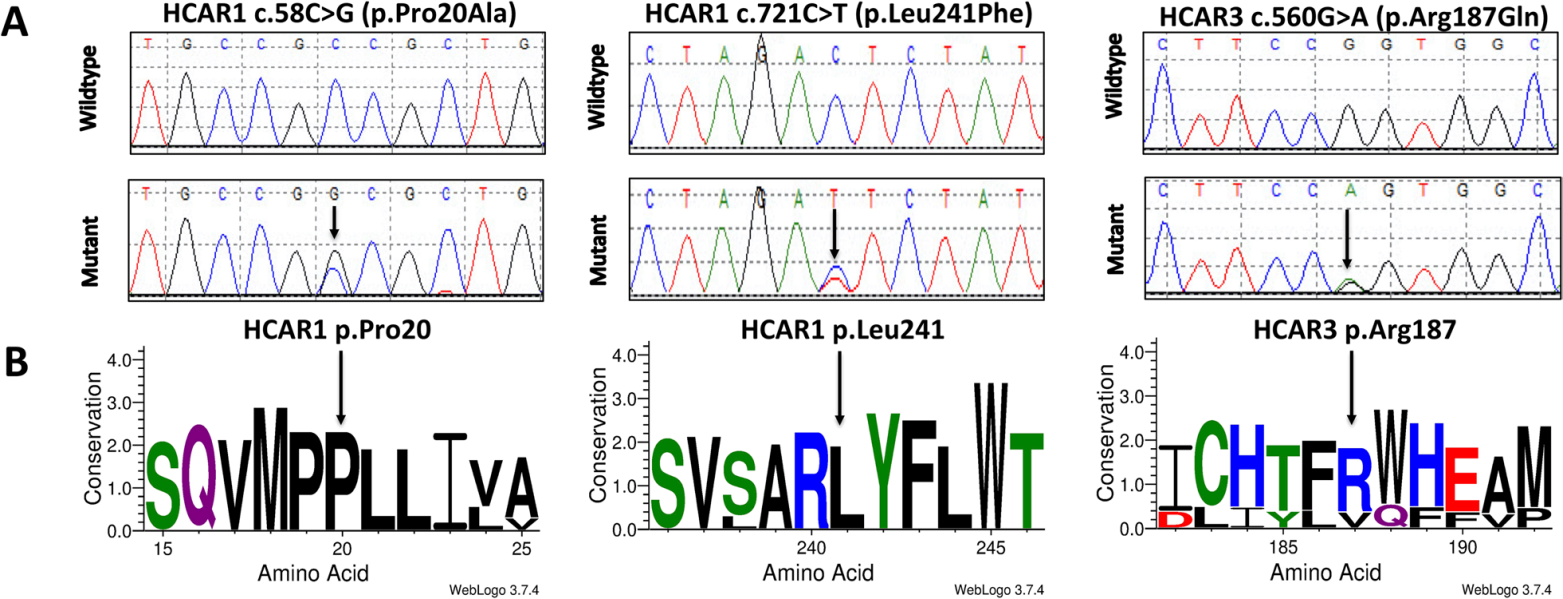
Rare missense variants in HCAR1 and HCAR3 detected in the AHCC. **A** Sanger sequencing comparisons of wildtype and mutant for HCAR1 variants p.P20A and p.L241F, and HCAR3 variant p.R187Q. **B** WebLogo amino acid conservation. The y-axis shows relative sequence conservation and the height of symbols in the stack show relative frequency for that position (HCAR1 was aligned with the following orthologues: Homo sapiens (Q9BXC0), Mus musculus (Q8C131), Canis lupus familiaris (B9UM26), Pan troglodytes (G2HJ56), Pongo pygmaeus (A0A4Y1JWL9), and Pan paniscus (A0A2R8ZEB9); HCAR3 was aligned with: Homo sapiens (P49019), Xenopus laevis (A0A1L8I0Z3), Pan troglodytes (H2RAM9), Pongo abelii (H2NJ01), Pan paniscus (A0A4Y1JWN7), and Pongo pygmaeus (A0A4Y1JWR4))

**Fig. 2:**
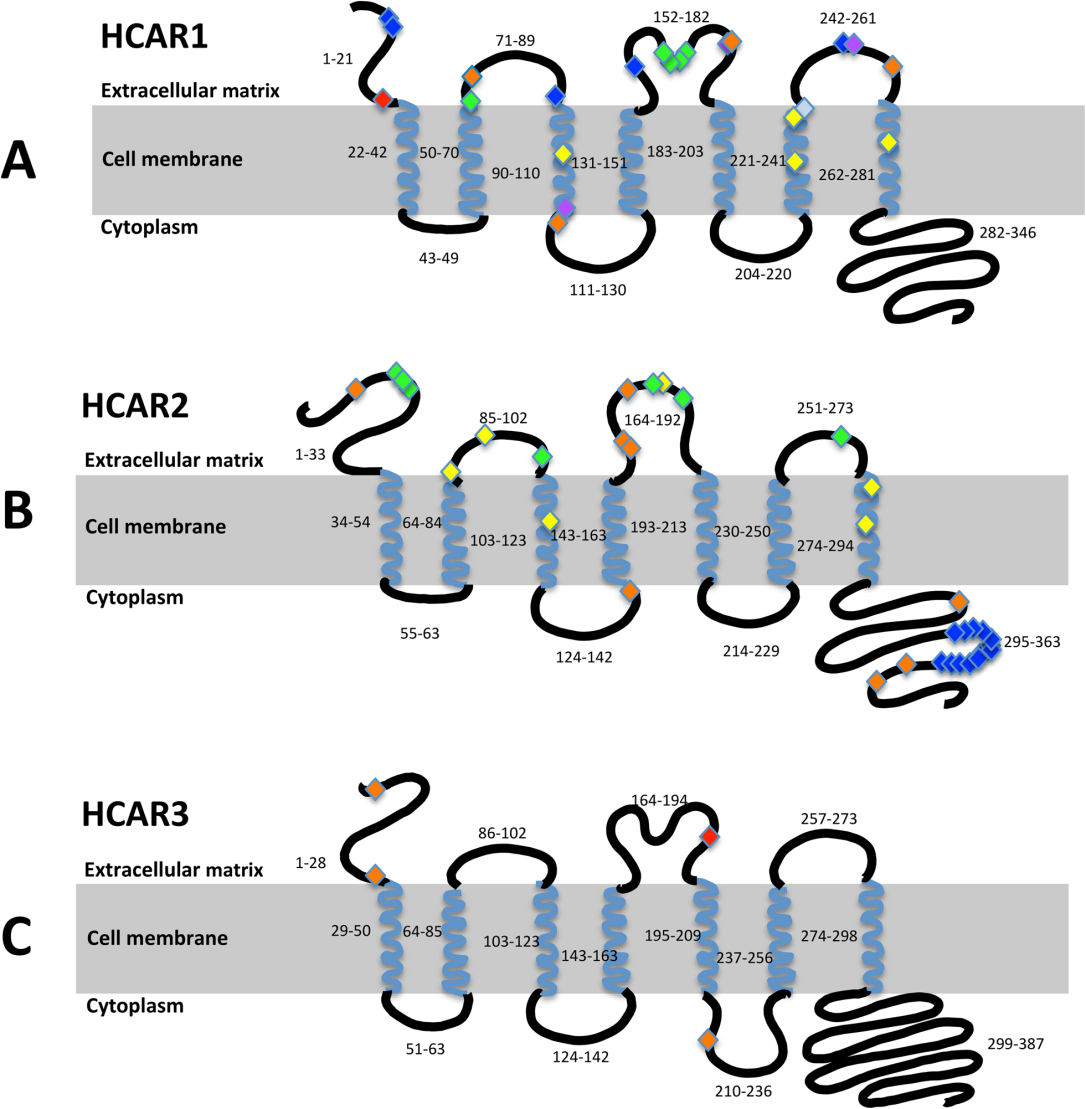
Topological illustration of HCAR1, HCAR2 and HCAR3 highlighting missense variants identified in the AHCC, TCGA, and previous functional studies. **A** HCAR1 (Q9BXC0); red: HCAR1 p.P20A detected in a BC case from the AHCC; orange: HCAR1 missense variants detected in TCGA BC cases, including p.Y74C, p.D112N, p.S172L, p.H257R; light blue/silver: p.L241F detected in both TCGA and AHCC BC cases; yellow: HCAR1 transmembrane domain variants, p.R99A, p.Y233A, p.R240A, and p.T267A, reported by Liu et al. to diminish the response to L-lactate [33]; green: HCAR1 residue R71 and motif C165-E166-S167-F168 identified by Kuei et al. as being critical for protein function [31]; blue: HCAR1 extracellular Cys residues, C6, 7, 88, 157, 165 (green) and 252, reported by Kuei et al. to abolish receptor activity when substituted with alanine or serine [31]; purple: HCAR1 variants p.A110V, p.S172L (covered by orange since it overlaps with variant detected in TCGA), and p.D253H identified by Doyle et al. as loss-of-function variants [32]. **B** HCAR2 (Q8TDS4); orange: HCAR2 missense variants detected in TCGA BC cases, including TCGA variants p.L11P, p.R142W, p.M167L, p.P168L, p.G173T, p.R311H, p.M346V, and p.G350S; yellow: N86/W91, R111, S178, F276, and Y284 were reported by Tunaru et al. as critical for binding of nicotinic acid [29]; green: Yasuda et al. identified an atypical motif N17-C18-C19 that is crucial for surface trafficking. They also identified C100, C177, C183, and C266, in the extracellular regions, which are important for HCAR2 activation [30]; blue: Li et al. identified a sequence of residues from 329–343 that plays a crucial role in keeping HCAR2 in an inactive conformation [28]. **C** HCAR3 (P49019); red: HCAR3 p.R187Q identified in a BC case in the AHCC; orange: HCAR3 missense variants detected in TCGA BC cases, including p.R3W, p.A27V, and p.R216W

**Fig. 3:**
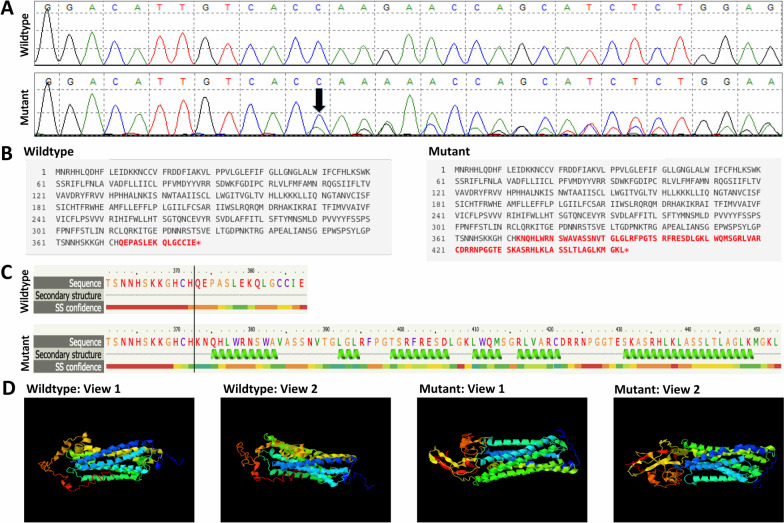
HCAR3 frameshift mutation, c.1117delC (p.Q373Kfs*82). **A** Sanger sequencing comparisons of wildtype and mutant, illustrating the deletion of the cytosine. **B** Mutalyzer comparison of the HCAR3 wildtype and mutant proteins, highlighting the different C-termini in red. **C** Phyre2 protein prediction software displaying secondary structure differences between wildtype and mutant. **D** I-TASSER protein prediction comparisons of the wildtype and mutant protein (each in two different views) to show the C-terminus extension and difference in tertiary structures; PyMOL was used to display the molecular graphics

A total of four rare, non-synonymous variants were identified in four different BC cases from the AHCC, two in *HCAR1* and two in *HCAR3* (Table 1 and Supplementary information: Table S4). In *HCAR1*, two highly conserved and probably damaging missense variants, *HCAR1* c.58C > G (p.P20A) and *HCAR1* c.721C > T (p.L241F), were identified (Table 1, and Figs. 1, 2A). *HCAR1* c.58C > G (p.P20A) had a statistically significant difference in allele frequencies between cases and controls, suggesting an association with BC risk (Table 1). Both *HCAR3* variants, *HCAR3* c.560G > A (p.R187Q) and *HCAR3* c.1117delC (p.Q373Kfs*82), also appeared to be associated with BC (Table 1). However, *HCAR3* c.560G > A (p.R187Q), located in an extracellular loop, was predicted to be benign (Table 1, Figs. 1, 2C, and Additional file 1: Figure S4). *HCAR3* c.1117delC (p.Q373Kfs*82) greatly extends the C-terminus of HCAR3 and changes the secondary and tertiary protein structure (Fig. 3). Moreover, PSORT predicted that the mutant HCAR3 p.Q373Kfs*82 protein loses a prenylation motif and gains both an ER Membrane Retention Signal and a peroxisomal targeting signal (Table 2).

**Table 1:** *HCAR* rare non-synonymous variants detected in the AHCC

Gene	GRCh38 position	rs ID	Alleles	GVS function	cDNA change	Protein change	MAF (%)		*p* Value	Polyphen2 (Missense variants only)
							AHCC BC cases	EVS controls		
HCAR1	chr12:122729619	rs140482291	G > A	missense	c.721C > T	p.L241F	1.087	0.151	0.139	Probably Damaging
	chr12:122730282	rs148912167	G > C	Missense	c.58C > G	p.P20A	1.087	0.000^	0.011	Probably Damaging
HCAR3	chr12:122715621	N/A	G > –	frameshift	c.1117delC	p.Q373Kfs*82	1.087	0.012	0.022	–
	chr12:122716178	rs373069919	C > T	missense	c.560G > A	p.R187Q	1.087	0.012	0.022	Benign

^Not detected in EVS; therefore, used "# of EA Samples Covered"

**Table 2:** PSORT predictions of HCAR3 wildtype and mutant, p.Gln373Lysfs*82

HCAR3 protein	ER Membrane Retention Signals:	NUCDISC: discrimination of nuclear localization signals	Tripeptide SKL-motif	Prenylation Motif	k-NN (k-nearest neighbors algorithm)			
Wildtype	KKXX-like motif in the C-terminus: NONE	content of basic residues: 9.8%	peroxisomal targeting signal in the C-terminus: NONE	CCIE	55.6%: endoplasmic reticulum	22.2% vacuolar	11.1% nuclear	11.1% Golgi
Mutant	KKXX-like motif in the C-terminus: KMGK	content of basic residues: 11.5%	peroxisomal targeting signal in the C-terminus: GKL	NONE	77.8%: endoplasmic reticulum	11.1% vacuolar	0.0% nuclear	11.1% Golgi

**Fig. 4:**
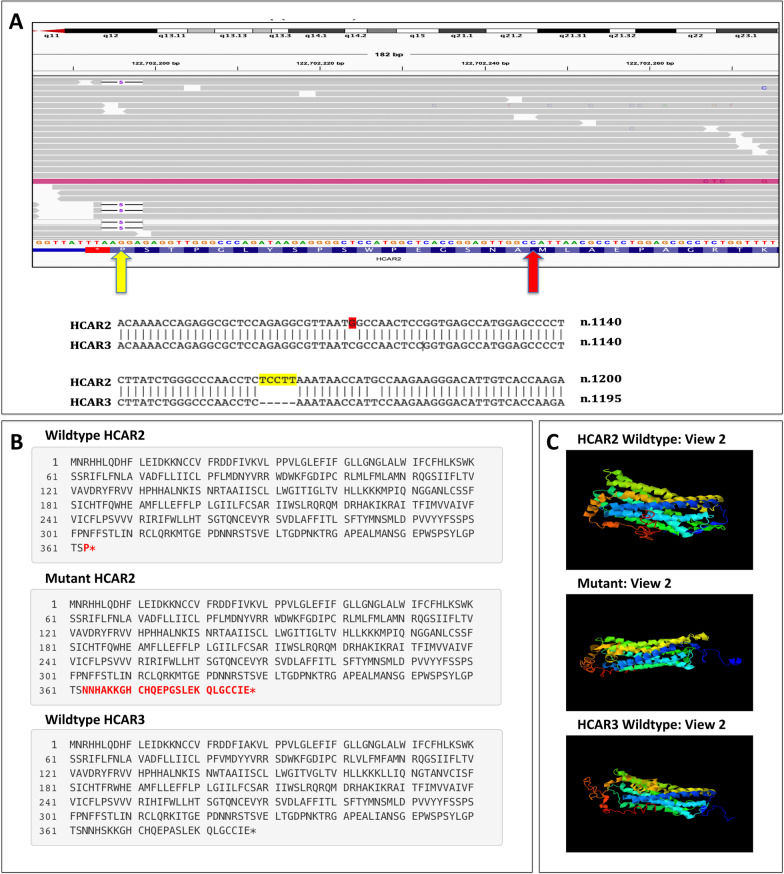
HCAR2 frameshift mutation, c. 1086_1090del (p.P363Nfs*26). **A** IGV screenshot (with sequences in the reverse complement) and HCAR2/3 sequence alignment displaying the locations of the mutation (yellow) and an additional HCAR2/3 sequence difference (red). **B** Mutalyzer comparison of the HCAR2 wildtype, HCAR2 mutant, and HCAR3 wildtype proteins, highlighting the different C-termini in red. **C** I-TASSER protein prediction comparisons of the HCAR2 wildtype, HCAR2 mutant, and HCAR3 wildtype proteins

Subsequent analysis of 649 BC cases in TCGA identified numerous rare, non-synonymous variants in *HCAR1*, *HCAR2,* and *HCAR3* (Table 3 and Fig. 2). No variants in *HCAR1* or *HCAR3* were associated with BC risk, including *HCAR1* c.721C > T (p.L241F), which was also detected in the AHCC, as well as other probably damaging variants (Table 3). Four BC-associated variants in *HCAR2* were identified, including one frameshift, *HCAR2* c.1086_1090del (p.P363Nfs*26) (Table 3 and Fig. 4), and three missense variants, *HCAR2* c.424C > T (p.R142W), *HCAR2* c.517_518delinsAC (p.G173T), and *HCAR2* c.1036A > G (p.M346V) (Table 3 and Figs. 2B). Aggregation analyses did not reveal any gene-based associations (Table 3 and Supplementary Information: Table S5).

**Table 3:** *HCAR* rare non-synonymous variants detected in TCGA

Gene	GRCh38 Position	rs ID	Alleles	GVS Function	cDNA Change	Protein Change	Polyphen2 (missense variants)	TCGA			EVS			Single variant P-values	Gene based aggregation analysis—non-synonymous variants
								EA Minor Allele #	EA Major Allele #	MAF (%) (EA)	EA Minor Allele #	EA Major Allele #	MAF (%) (EA)		
HCAR1	12:122729570	rs141008238	T > C	missense	c.770A > G	p.H257R	Benign	1	1295	0.08	0	8600	0.00	0.131	1
	12:122729619	rs140482291	G > A	missense	c.721C > T	p.L241F	Probably damaging	4	1292	0.31	13	8587	0.15	0.266	
	12:122729825	rs61742326	G > A	missense	c.515C > T	p.S172L	Benign	6	1290	0.46	31	8569	0.36	0.622	
	12:122730006	rs749553547	C > T	missense	c.334G > A	p.D112N	Probably damaging	1	1295	0.08	0	8600	0.00	0.131	
	12:122730119	rs201991947	T > C	missense	c.221A > G	p.Y74C	Probably damaging	2	1294	0.15	5	8595	0.06	0.231	
HCAR2	12:122702194	rs63475561	AAGGA > -	frameshift	c.1086_1090del	p.P363Nfs*26	-	2	1294	0.15	0	8600	0.00	0.017	0.238
	12:122702236	rs148160325	C > T	missense	c.1048G > A	p.G350S	Benign	1	1295	0.08	1	8599	0.01	0.131	
	12:122702248	rs145934041	T > C	missense	c.1036A > G	p.M346V	Benign	3	1293	0.23	1	8599	0.01	0.002	
	12:122702352	rs780477417	C > T	missense	c.932G > A	p.R311H	Probably damaging	1	1295	0.08	0	8600	0.00	0.131	
	12:122702766	rs144376493	C > G	missense	c.517_518delinsAC	p.G173T	Benign	4	1292	0.31	0	8600	0.00	2.93e-4	
	12:122702781	rs145014727	G > A	missense	c.503C > T	p.P168L	Benign	4	1292	0.31	26	8574	0.30	1	
	12:122702785	rs147573131	T > A	missense	c.499A > T	p.M167L	Benign	4	1292	0.31	28	8572	0.33	1	
	12:122702860	rs151172149	T > C	missense	c.424C > T	p.R142W	Benign	3	1293	0.23	1	8599	0.01	0.002	
	12:122703252	rs755259236	C > T	missense	c.32 T > C	p.L11P	Benign	1	1295	0.08	1	8599	0.01	0.131	
HCAR3	12:122716092	rs200839014	G > A	missense	c.646C > T	p.R216W	Possibly damaging	1	1295	0.08	0	8600	0.00	0.131	1
	12:122716658	rs148858491	G > A	missense	c.80C > T	p.A27V	Benign	4	1292	0.31	12	8588	0.14	0.148	
	12:122716731	rs190370423	G > A	missense	c.7C > T	p.R3W	Benign	1	1295	0.08	1	8599	0.01	0.245

## Discussion

Upon screening 46 European American BC cases from the AHCC for rare, non-synonymous variants in *HCAR1*, *HCAR2*, and *HCAR3*, a total of four variants were identified in four different BC cases. These variants were exclusively identified in *HCAR1* and *HCAR3*, which is notable considering their suggested oncogenic role and requirement for BC proliferation/survival compared to the demonstrated tumor suppressor properties of *HCAR2* [4–6]. Subsequent screening of a larger cohort, 649 European American BC cases from TCGA, identified numerous rare, non-synonymous variants in *HCAR1*, *HCAR2,* and *HCAR3*, but only variants in *HCAR2* were associated with BC risk. No gene-based associations were identified.

*HCAR2*, the nicotinic acid receptor, has recently been reported to have BC tumor suppressing properties [4, 5]; thus, rare, non-synonymous variants in HCAR2 likely disrupt protein function, increasing BC susceptibility. No *HCAR2* variants were detected in the AHCC. This was likely due to the small cohort size and the rarity of such variants. Nine different *HCAR2* variants were detected in the TCGA BC cohort, four of which generated statistically significant p-values. One statistically significant variant was *HCAR2* c.1086_1090del (p.P363Nfs*26). This five base pair deletion affects one of the few differences between the nucleotide sequences of *HCAR2* and *HCAR3* (Fig. 4). Typically, *HCAR2* has those five nucleotides, which *HCAR3* lacks. Because of this extreme homology and the possibility of misalignment of *HCAR3* reads, variant confirmation was pertinent. Ultimately, this variant and all other reported *HCAR2* variants were confirmed through visualization in IGV and the comparison of proximate *HCAR2/3* differences within the same mutation-harboring reads (Table 3 and Additional file 1: Figure S5). This process also eliminated variants that were a result of misalignment (Additional file 1: Figure S7). Ultimately, the HCAR2 p.P363Nfs*26 mutant protein was predicted to gain 25 amino acids at its C-terminus, mimicking the C-terminus of HCAR3 (Fig. 4). This conversion could explain the variant’s pathogenicity since the HCAR2 C-terminus is essential for normal function [28], and *HCAR3* has BC oncogenic properties [5].

Several HCAR2 residues have been previously demonstrated to be critical for protein function (Fig. 2B) [28–30]. Tunaru et al*.* reported N86, W91, R111, S178, F276, and Y284 as critical for nicotinic acid binding [29]. Yasuda et al*.* identified an atypical motif, N17-C18-C19, crucial for normal surface trafficking and other residues in extracellular regions, C100, C177, C183, and C266, essential for HCAR2 activation [30]. Furthermore, Li et al. discovered a sequence of residues (329–343) in the C-terminus critical in keeping HCAR2 in an inactive conformation [28]. Our study did not identify *HCAR2* missense variants that specifically affected the aforementioned critical residues, but some identified variants were in close proximity (Fig. 2B). Interestingly, most of the detected *HCAR2* missense variants were predicted to be benign, including the three BC-associated missense variants, *HCAR2* c.424C > T (p.R142W), *HCAR2* c.517_518delinsAC (p.G173T), and *HCAR2* c.1036A > G (p.M346V). Similar to *HCAR2* c.1086_1090del (p.P363Nfs*26), both *HCAR2* c.424C > T (p.R142W) and *HCAR2* c.517_518delinsAC (p.G173T) affect nucleotides that differ between *HCAR2* and *HCAR3*, yet they too were confirmed as true variant calls (Additional file 1: Figure S5). These missense variants could convert HCAR2’s function since they result in residues specific to HCAR3. *HCAR2* c.424C > T (p.R142W) is located at a transmembrane/cytoplasmic loop junction. Notably, several critical residues in HCAR2 [29, 30] and HCAR1 [31, 32] are at or near such junctions (Fig. 2A, B).

The HCAR1 protein is known as the lactate receptor, and, upon HCAR1 binding, lactate inhibits lipolysis [1, 2, 33]. Like HCAR2, many residues have been deemed critical for HCAR1 function (Fig. 2A) [31–33]. Most notably, *HCAR1* c.515C > T (p.S172L), a loss-of-function variant identified by Doyle et al*.* [32], was detected in six BC cases in the TCGA cohort. Interestingly, PolyPhen2 analysis predicted the variant to be benign, demonstrating the potential inaccuracies of such prediction software [34]. Furthermore, despite being detected at a higher frequency in BC cases than controls, the difference was not statistically significant; thus, additional studies are required to determine if this loss-of-function variant is associated with BC risk. Regarding other critical residues, Liu et al*.* demonstrated that particular missense variants in transmembrane domains three (p.R99A), six (p.Y233A and p.R240A), and seven (p.T267A) diminished the response of HCAR1 to L-lactate (Fig. 2A) [33]. It is important to note the proximity of p.R240A to another missense variant detected in this study, p.L241F, which is at a transmembrane and extracellular domain junction (Fig. 2A). The vital function of R240 in lactate binding, as well as the identification of other critical residues located at or near similar junctions (Fig. 2A) [31, 32], hints towards the importance of the highly conserved L241 and the potentially damaging effects of an amino acid substitution at that location. Interestingly, HCAR1 p.L241F was the only variant detected in both BC cohorts (AHCC and TCGA) in this study. Despite not appearing to be associated with BC, it is a rare allele that is predicted to be damaging. Coincidentally, two other *HCAR1* missense variants detected in this study, *HCAR1* c.58C > G (p.P20A) and *HCAR1* c.334G > A (p.D112N), are also near a transmembrane and extracellular/cytoplasmic domain junction (Fig. 2A). Furthermore, HCAR1 p.P20A is in the N-terminus close to two other critical cysteine residues, C6 and C7 (Fig. 2A) [31]. The N-terminus is essential for ligand binding, dimerization, signaling, and surface expression (31, 35–37). Therefore, HCAR1 p.P20A is likely a functional variant. It was also determined to be associated with BC in the AHCC cohort.

Considering that five of the seven detected HCAR1 missense variants are predicted to be pathogenic, determining their functional involvement in BC is critical. To date, all functionally assessed *HCAR1* variants have been deemed loss-of-function (highlighted in color in Fig. 2A) [31–33]. However, with HCAR1 typically being regarded as critical for BC proliferation and survival by controlling lipid/fatty acid metabolism [5, 6], a loss-of-function mutation would presumably result in BC cell death. Interestingly, knocking down *HCAR1* has different effects on different BC molecular subtypes [5, 6]. For instance, knocking down *HCAR1* in a HER2-enriched BC cell line, HCC1954 (ER-, PR-, HER2 +), and triple-negative BC cell line, HCC38 (ER-, PR-, HER2-), resulted in a significant decrease of cell viability within 48 h of transfection. However, there was no significant change in viability regarding the luminal B cell line, BT-474 (ER + , PR + , HER2 +), similar to the non-tumorigenic epithelial breast cell line, MCF12A [5]. Furthermore, when *HCAR1* was knocked down in the luminal A BC cell lines, MCF-7 and T47D, cell viability decreased [6]. In this study, the BC molecular subtype was available for most BC cases in the AHCC; thus, we confirmed that the individuals in the AHCC with HCAR1 p.P20A and HCAR1 p.L241F were diagnosed with luminal subtypes. Specifically, the individual with HCAR1 p.P20A had luminal A BC (ER + and HER2-), which according to Lee et al*.* requires HCAR1 to proliferate [6]. The individual with HCAR1 p.L241F was ER + and PR + , but HER2 status was unknown; thus, the subtype could not be confirmed as luminal A or B. If it were luminal B, *HCAR1* expression would not be not required for survival [5], whereas it would be necessary for luminal A [6]. In addition to knock-down studies, Lee et al*.* investigated *HCAR1* expression levels in different BC molecular subtypes and noted that ER + BC cell lines expressed *HCAR1* at a higher level [6]; this ER + BC-association has also been reported for another GPCR, GPR30 [38]. Thus, it is important to determine if *HCAR1* missense variants are specifically associated with ER + BC, as well as if they are loss- or gain-of-function mutations. Unfortunately, the sample size of the AHCC was too small to determine subtype-specific associations, and subtype information was not provided in the clinical information of the TCGA dataset.

HCAR3, which is only found in higher primates, is the receptor for 3-hydroxylated β-oxidation intermediates, particularly 3-hydroxy-octanoate [1, 2]. When activated, HCAR3 inhibits free fatty acid release from cells, providing a negative feedback mechanism to offset stimuli that promote lipolysis and fatty acid oxidation. Knocking down *HCAR3* in BC cell lines BT-474, HCC1954, and HCC38 induced cell death, suggesting that HCAR3 has oncogenic properties. Introducing fatty acid oxidation inhibitors mitigated the knock-down effects, confirming that uncontrolled up-regulation of fatty acid oxidation promotes BC cell death; thus, HCAR3 plays a vital role in controlling fatty acid metabolism in BC cells [5]. Accordingly, one can presume that BC-associated *HCAR3* variants have gain-of-function effects. However, *HCAR3* knock-down effects have not been assessed in luminal A BC cell lines, which is the molecular subtype reported in the two BC-affected individuals from the AHCC with *HCAR3* BC-associated variants, *HCAR3* c.560G > A (p.R187Q) and *HCAR3* c.1117delC (p.Q373Kfs*82). Numerous *HCAR3* genetic variants have been reported in publically available databases [13, 39], as well as through polymerase chain reaction (PCR)-based techniques [40]. While their pathogenic effects have not been functionally assessed, screening results of the AHCC suggested that rare, non-synonymous variants in *HCAR3* may enhance the receptor’s ability to control fatty acid metabolism. Nonetheless, three *HCAR3* missense variants detected in BC cases in TCGA did not appear to be associated with BC risk. Additionally, HCAR3 p.R187Q was predicted to be benign. Even though prediction software have been shown to misclassify known pathogenic variants [34], it is important to note that HCAR2 has a glutamine at that overlapping position. Though we have confirmed HCAR3 p.R187Q through nested PCR (Additional file 1: Figure S4), it is unknown if this change would affect the function of HCAR3. That being said, with such slight differences between the two proteins, perhaps each alteration is key to protein function. Incidentally, this variant was detected in an early onset BC case also determined to harbor a clinically significant frameshift mutation in *NBN* [12, 41]. The interaction of these two variants and their combined ability to promote BC is unknown, but, intriguingly, expression of both *HCAR3* and *NBN* have been reported to be dysregulated in the oocytes of older women, when investigating why aneuploidy pregnancies occur in women of older ages. Overall, this observation suggests that these genes may play a role in proper chromosome segregation and maintaining genomic integrity, which is a phenomenon also disrupted in cancer [42, 43].

The *HCAR3* frameshift mutation, HCAR3 p.Q373Kfs*82, significantly extends the C-terminus cytoplasmic tail of the mutant HCAR3 protein and changes the secondary and tertiary protein structure (Fig. 3). Again, based on the suggested oncogenic role of HCAR3 in BC, HCAR3 p.Q373Kfs*82 may potentially result in a gain-of-function. Interestingly, distinct mutation profiles, corresponding to clusters of nonsense and frameshift mutations in the C-termini of GPCRs, GPR34, CCR6, and CCR4, have been reported in mucosa-associated lymphoid tissue (MALT) lymphoma and adult T cell leukemia/lymphoma (ATLL) as gain-of-function mutations [44–46]. Even though the nonsense and frameshift mutations reported in *GPR34*, *CCR6*, and *CCR4* truncate the encoded proteins, PSORT predicted that HCAR3 p.Q373Kfs*82 abolishes a prenylation motif. Similarly, the *GPR34* mutations eliminate a key phosphorylation motif and ultimately dysregulate the receptor’s desensitization process [44, 45]. Additionally, the mutant HCAR3 protein gains an ER Membrane Retention Signal, potentially affecting internalization patterns, which is also disrupted with *CCR4* gain-of-function mutations [44, 46]. Contrarily, the gain of a predicted peroxisomal targeting signal to the mutant HCAR3 hints toward protein degradation, a loss-of-function mechanism. On a similar note, read-through mutations that result in mutant proteins with C-terminal extensions in *PNPO* and *HSD3B2* cause hereditary disorders through protein degradation [47]. Nonetheless, *GATA3* frameshift mutations that extend the C-terminus are the most common somatic mutation identified in TCGA BC patients and display gain-of-function activity [48]. In addition, loss-of-function *GATA3* mutations were also identified, demonstrating that both loss- and gain-of-function mutations can be identified in the same gene and associated with BC. Similarly, *TP53*, a clinically valid BC susceptibility gene, has both tumor suppressor and oncogenic properties [49–51]. Thus, the exact functional consequences of HCAR3 p.Q373Kfs*82 may be complex but are important to elucidate, especially considering the vital functions of the C-terminus of HCAR proteins [28].

## Conclusions

*HCAR1*, *HCAR2*, and *HCAR3* are three genes clustered on chromosome 12 that encode HCARs, known GPCRs that play a critical role in lipid metabolism, even in the context of BC proliferation and survival. Upon genetic analysis of two cohorts of BC cases, potentially damaging, non-synonymous genetic variants in *HCAR1*, *HCAR2*, and *HCAR3* were identified that could alter receptor function. Though no gene-based associations were revealed, the identification of individual variant associations supports the need to investigate the functional consequences of these variants. However, these genetic associations need to be validated in future studies. Ultimately, it is vital to understand how these genetic variants play a role in BC risk and proliferation and their consequences on treatment strategies, particularly regarding the use of doxorubicin, a commonly prescribed BC chemotherapy drug [7, 8].

## Supplementary Information

**Additional file 1**. Details on primers, nested PCRs, and variant confirmation.

## Data Availability

Sanger sequencing data of HCAR breast cancer mutations are available through two different respositories; fasta files can be obtained through the NCBI SRA repository through BioProject PRJNA778760 (https://www.ncbi.nlm.nih.gov/sra/PRJNA778760); ab1 files can be obtained through the Auburn University Scholarly Repository (Aurora; https://doi.org/10.35099/aurora-83). TCGA whole-exome sequencing data analyzed during this study are available for download through the NCI GDC Data Portal (https://portal.gdc.cancer.gov). See methods for specific repository filters used for sample acquisition from the TCGA-BRCA project.

## Abbreviations

AHCC: Alabama Hereditary Cancer Cohort
ATLL: Adult T cell leukemia/lymphoma
BAM: Binary sequence alignment mapping
BC: Breast cancer
DCIS: Ductal carcinoma in situ
ER: Estrogen receptor
EVS: Exome Variant Server
GDC: Genomic Data Commons
GPCR: G-protein coupled receptor
HCAR: Hydroxycarboxylic acid receptor
HER2: Human epidermal growth factor receptor 2
IGV: Integrative Genomic Viewer
MAF: Minor allele frequency
MALT: Mucosa-associated lymphoid tissue
NB: Blood-Derived Normal
PCR: Polymerase chain reactions
PR: Progesterone receptor
SIFT: Sorting intolerant from tolerant
TCGA: The Cancer Genome Atlas
VCF: Variant calling format
