# Supplementary material for: Rare and potentially pathogenic variants in hydroxycarboxylic acid receptor genes identified in breast cancer cases
Source: BMC Med Genomics. 2021 Dec 1;14:284. doi: 10.1186/s12920-021-01126-3 (PMC8638184; doi:10.1186/s12920-021-01126-3)
Supplement: Supplementary file 1 — Additional file 1. Details on primers, nested PCRs, and variant confirmation. [file 12920_2021_1126_MOESM1_ESM.pdf]

Table S1: HCAR1 primers for nested PCR

HCAR1 (NM_032554.3) Nested PCR	Primer name	Primer direction	Primer sequence	% GC	Tm (˚C)	Amplicon size (bp)	Genomic position of amplicon (GRCh38/hg38)
External primers for initial PCR	NM_032554.3_Ex1a_F	Forward	CCTACCGCCTCTCAGAAATG	55	59.8	3141	chr12:122727524-122730664
	NM_032554.3_Ex1f_R	Reverse	ATGGCACGATGTCACCTCAC	55	62.0		
Internal primer set A*	NM_032554.3_Ex1a_F	Forward	CCTACCGCCTCTCAGAAATG	55	59.8	531	chr12:122730134-122730664
	NM_032554.3_Ex1a_R	Reverse	GGCAGGCAGATCATAAGGAG	55	59.8		
Internal primer set B*	NM_032554.3_Ex1b_F	Forward	CTGGGCGCACTAGGCAAT	61	62.3	599	chr12:122729660-122730258
	NM_032554.3_Ex1b_R	Reverse	ATTGCCACCACCATGATGAA	45	62.2		
Internal primer set C*	NM_032554.3_Ex1c_F	Forward	CCCTCGGCATCATCTTATTTT	43	60.3	591	chr12:122729187-122729777
	NM_032554.3_Ex1c_R	Reverse	AAGCCGTCTTGCAATAAGAAA	38	59.0		
Internal primer set D	NM_032554.3_Ex1d_F	Forward	GCACTGAACAAGCAGACCAA	50	60.0	600	chr12:122728706-122729305
	NM_032554.3_Ex1d_R	Reverse	CCTGTGATCCCACCACTTTG	55	61.4		
Internal primer set E	NM_032554.3_Ex1e_F	Forward	TTTTTAGTAGAGACAGGGTTTTGC	38	58.2	600	chr12:122728210-122728809
	NM_032554.3_Ex1e_R	Reverse	CAATTCACAGTCACATGACAAGTT	38	59.1		
Internal primer set F	NM_032554.3_Ex1f_F	Forward	CAATCTGCTCTGACCTTCTTCA	46	59.6	838	chr12:122727524-122728361
	NM_032554.3_Ex1f_R	Reverse	ATGGCACGATGTCACCTCAC	55	62.0		

*internal primers that covered coding sequence and were used in this study for mutation analysis

Table S2: HCAR2 primers for nested PCR

HCAR2 (NM_177551.3) Nested PCR	Primer name	Primer direction	Primer sequence	% GC	Tm (˚C)	Amplicon size (bp)	Genomic position of amplicon (GRCh38/hg38)
External primer set for initial PCR	NM_177551.3_Ex1a_F	Forward	CTACAAGGCGGGGTTCCATG	60	64.8	4343	chr12:122700573-122704915
	NM_177551.3_Ex1h_R	Reverse	TCCTTTCCTTCTCTGAATTGGGA	44	63.4		
Internal primer set A	NM_177551.3_Ex1a_F	Forward	CTACAAGGCGGGGTTCCATG	60	64.8	728	chr12:122704188-122704915
	NM_177551.3_Ex1a_R	Reverse	AGCTGTAGCAAATCGGGTGT	50	59.8		
Internal primer set B	NM_177551.3_Ex1b_F	Forward	CCCCATGCTCTATCCTAAGGT	52	59.5	700	chr12:122703579-122704278
	NM_177551.3_Ex1b_R	Reverse	CGACTGTCATTCGAAAATCAGTGA	42	63.6		
Internal primer set C*	NM_177551.3_Ex1c_F	Forward	TGTCAGCCAAGCAGATGACG	55	63.6	693	chr12:122703006-122703698
	NM_177551.3_Ex1c_R	Reverse	CAGTCCCAACGCCTCACATA	55	62.1		
Internal primer set D*	NM_177551.3_Ex1d_F	Forward	GCCTTGCCCTGTGGATTTTC	55	64.0	696	chr12:122702452-122703147
	NM_177551.3_Ex1d_R	Reverse	TAAAGAACGCCAGGTCCACC	55	62.3		
Internal primer set E*	NM_177551.3_Ex1e_F	Forward	AGCGTGGTTGTGCGGATC	61	63.3	696	chr12:122701850-122702545
	NM_177551.3_Ex1e_R	Reverse	TGTGGGAAGAAGGCCAACAA	50	63.8		
Internal primer set F	NM_177551.3_Ex1f_F	Forward	GCAGGACTGAAGATGGGCAA	55	63.6	700	chr12:122701241-122701940
	NM_177551.3_Ex1f_R	Reverse	AAGTAAGAAGTCCATTTCTGTGAAC	36	57.2		
Internal primer set G	NM_177551.3_Ex1g_F	Forward	CAGTTAGAGGGGTGCACGTT	55	60.2	526	chr12:122700860-122701385
	NM_177551.3_Ex1g_R	Reverse	GTAGAAGTGGGGTTTCGCCA	55	62.3		
Internal primer set H	NM_177551.3_Ex1h_F	Forward	AGAGACACAGACACTGAGCT	50	52.2	600	chr12:122700573-122701172
	NM_177551.3_Ex1h_R	Reverse	TCCTTTCCTTCTCTGAATTGGGA	44	63.4		

*internal primers that covered coding sequence and were used in this study for mutation analysis

Table S3: HCAR3 primers for nested PCR

HCAR3 (NM_006018.2) Nested PCR	Primer name	Primer direction	Primer sequence	% GC	Tm (˚C)	Amplicon size (bp)	Genomic position of amplicon (GRCh38/hg38)
External primer set for initial PCR	NM_006018.2_Ex1a_F	Forward	ACACTCTCCAGCAGGCTTTT	50	59.1	3199	chr12:122714250-122717448
	NM_006018.2_Ex1f_R	Reverse	TTCTCCCAGCCTTCCCAGTA	55	62.0		
Internal primer set A	NM_006018.2_ Ex1a_F	Forward	ACACTCTCCAGCAGGCTTTT	50	59.1	658	chr12:122716791-122717448
	NM_006018.2_Ex1a_R	Reverse	AGTGGCGTGTGTCTGTATGG	55	59.6		
Internal primer set B*	NM_006018.2_Ex1b_F	Forward	AGTCATCTATTTCAACACCCTGACA	40	61.1	598	chr12:122716271-122716868
	NM_006018.2_Ex1b_R	Reverse	ACAGTGATGCCCCACAGAAG	55	61.1		
Internal primer set C*	NM_006018.2_Ex1c_F	Forward	TATTTCCGGGTGGTCCATCC	55	64.0	600	chr12:122715763-122716362
	NM_006018.2_Ex1c_R	Reverse	GCGGTTATTATCTGGCTCACC	52.4	60.8		
Internal primer set D*	NM_006018.2_Ex1d_F	Forward	ACCCCGTGGTGTACTACTTCT	52.4	57.6	599	chr12:122715271-122715869
	NM_006018.2_Ex1d_R	Reverse	TATCACCCCAGGAGCTGAGC	60	62.6		
Internal primer set E	NM_006018.2_Ex1e_F	Forward	GAGCAGAGTTGGAGCCAGAG	60	60.3	622	chr12:122714742-122715363
	NM_006018.2_Ex1e_R	Reverse	TAGCAACGCCAGACTGTCTC	55	59.2		
Internal primer set F	NM_006018.2_Ex1f_F	Forward	GCTGTTTCCCACCTGTTTGC	55	62.9	654	chr12:122714250-122714903
	NM_006018.2_Ex1f_R	Reverse	TTCTCCCAGCCTTCCCAGTA	55	62.0		

*internal primers that covered coding sequence and were used in this study for mutation analysis

Table S4: Details for HCAR1 and HCAR3 rare non-synonymous variants detected in the AHCC.

Gene	GRCh38 Position	rs ID	Coding change	Protein change	AHCC BC Cases								EVS (European Americans)							
					Genotype				Number of alleles			MAF (%)	Genotype				Number of alleles			MAF (%)
					Het variant	Homo variant	Homo wt	Total	Minor allele	Major allele	Total		Het variant	Homo variant	Homo wt	Total	Minor allele	Major allele	Total	
HCAR1	chr12:122730282	rs148912167	c.58C>G	p.(P20A)	1	0	45	46	1	91	92	1.087	0	0	4298	4298^	0	8596	8596	0.011
	chr12:122729619	rs140482291	c.721C>T	p.(L241F)	1	0	45	46	1	91	92	1.087	13	0	4287	4300	13	8587	8600	0.139
HCAR3	chr12: 122716178	rs373069919	c.560G>A	p.(R187Q)	1	0	45	46	1	91	92	1.087	1	0	4299	4300	1	8599	8600	0.022
	chr12:122715620	N/A	c.1117delC	p.(Q373Kfs*82)	1	0	45	46	1	91	92	1.087	1	0	4126	4127	1	8253	8254	0.022

^not detected in EVS; therefore, used "# of EA Samples Covered".

Table S5: All rare, non-synonymous variants detected in TCGA and EVS used in the gene-based aggregation analyses (see next page).

Genes	GRCh38 Position	rs ID	Alleles	mRNA Accession #	GVS Function	cDNA Change	Protein Change	TCGA			EVS			Single Variant p-values	Gene based aggregation analysis – non-synonymous variants
								EA Minor Allele #	EA Major Allele #	MAF (%) (EA)	EA Minor Allele #	EA Major Allele #	MAF (%) (EA)		
HCAR1	12:122729360	rs376788646	G>C	NM_032554.3	missense	c.980C>G	p.(A327G)	0	1296	0	1	8599	0.015	1	1
	12:122729411	rs145500404	G>A	NM_032554.3	missense	c.929C>T	p.(P310L)	0	1296	0	1	8599	0.015	1	
	12:122729570	rs141008238	T>A	NM_032554.3	missense	c.770A>T	p.(H257L)	0	1296	0	1	8599	0.015	0.245	
	12:122729570	rs141008238	T>C	NM_032554	missense	c.770A>G	p.(H257R)	1	1295	0.077	0	8600	0	0.131	
	12:122729619	rs140482291	G>A	NM_032554.3	missense	c.721C>T	p.(L241F)	4	1292	0.309	13	8587	0.151	0.266	
	12:122729631	rs371072988	C>T	NM_032554.3	missense	c.709G>A	p.(V237M)	0	1296	0	2	8598	0.023	1	
	12:122729643	rs138206647	A>G	NM_032554.3	missense	c.697T>C	p.(Y233H)	0	1296	0	1	8599	0.013	1	
	12:122729673	rs368288293	T>A	NM_032554.3	missense	c.667A>T	p.(M223L)	0	1296	0	1	8599	0.012	1	
	12:122729682	rs372254985	G>C	NM_032554.3	missense	c.658C>G	p.(R220G)	0	1296	0	1	8599	0.012	1	
	12:122729693	unknown	R>A1	NM_032554.3	coding	c.646_647insTGA	p.(K216delinsMK)	0	1296	0	1	8249	0.012	1	
	12:122729700	rs149599946	G>A	NM_032554.3	missense	c.640C>T	p.(R214W)	0	1296	0	1	8599	0.012	1	
	12:122729825	rs61742326	G>A	NM_032554.3	missense	c.515C>T	p.(S172L)	6	1290	0.463	31	8569	0.361	0.622	
	12:122729853	rs139197891	C>T	NM_032554.3	missense	c.487G>A	p.(V163I)	0	1296	0	2	8598	0.023	1	
	12:122729907	rs140218785	G>C	NM_032554.3	missense	c.433C>G	p.(L145V)	0	1296	0	1	8599	0.012	1	
	12:122729934	rs201612647	C>A	NM_032554.3	missense	c.406G>T	p.(V136F)	0	1296	0	1	8599	0.012	1	
	12:122729951	rs370201170	C>G	NM_032554.3	missense	c.389G>C	p.(R130P)	0	1296	0	1	8599	0.012	1	
	12:122729987	rs376395828	A>G	NM_032554.3	missense	c.353T>C	p.(V118A)	0	1296	0	1	8599	0.012	1	
	12:122730006	rs749553547	C>T	NM_032554.4	missense	c.334G>A	p.(D112N)	1	1295	0.077	0	8600	0	0.131	
	12:122730011	rs61747158	G>A	NM_032554.3	missense	c.329C>T	p.(A110V)	0	1296	0	1	8599	0.012	1	
	12:122730039	rs139090131	C>T	NM_032554.3	missense	c.301G>A	p.(G101R)	0	1296	0	1	8599	0.012	1	
	12:122730060	rs199570616	T>G	NM_032554.3	missense	c.280A>C	p.(T94P)	0	1296	0	1	8599	0.012	1	
	12:122730119	rs201991947	T>C	NM_032554.3	missense	c.221A>G	p.(Y74C)	2	1294	0.154	5	8595	0.058	0.231	
	12:122730129	unknown	R>A1	NM_032554.3	frameshift	c.210del1	p.(R71Gfs*27)	0	1296	0	9	8243	0.109	0.380	
	12:122730152	rs139899793	A>G	NM_032554.3	missense	c.188T>C	p.(L63P)	0	1296	0	1	8599	0.012	1	
	12:122730165	rs143399382	C>T	NM_032554.3	missense	c.175G>A	p.(V59M)	0	1296	0	3	8597	0.035	1	
	12:122730293	rs377701759	T>A	NM_032554.3	missense	c.47A>T	p.(Q16L)	0	1296	0	1	8599	0.012	1	
HCAR2	12:122702194	rs63475561	AAGGA>-	NM_177551.3	frameshift	c.1086_1090del	p.(P363Nfs*26)	2	1294	0.154	**0**	**8600**	**0**	0.017	0.238
	12:122702236	rs148160325	C>T	NM_177551.3	missense	c.1048G>A	p.(G350S)	1	1295	0.077	1	8599	0.012	0.131	
	12:122702248	rs145934041	T>C	NM_177551.3	missense	c.1036A>G	p.(M346V)	3	1293	0.231	1	8599	0.012	0.002	
	12:122702293	rs138616040	G>A	NM_177551.3	missense	c.991C>T	p.(L331F)	0	1296	0	1	8599	0.012	1	
	12:122702304	rs143584617	G>A	NM_177551.3	missense	c.980C>T	p.(T327M)	0	1296	0	2	8598	0.023	1	
	12:122702352	rs780477417	C>T	NM_177551.3	missense	c.932G>A	p.(R311H)	1	1295	0.077	**0**	**8600**	**0**	0.131	
	12:122702367	unknown	R>A1	NM_177551.3	coding	c.914_916del3	p.(F305_S306delinsS)	0	1296	0	2	8252	0.024	1	
	12:122702492	rs370245808	C>A	NM_177551.3	missense	c.792G>T	p.(Q264H)	0	1296	0	1	8599	0.012	1	
	12:122702496	rs374201682	G>A	NM_177551.3	missense	c.788C>T	p.(T263M)	0	1296	0	1	8599	0.012	1	
	12:122702609	rs201310632	C>A	NM_177551.3	missense	c.675G>T	p.(K225N)	0	1296	0	1	8557	0.012	1	
	12:122702638	rs142084430	G>A	NM_177551.3	missense	c.646C>T	p.(R216W)	0	1296	0	1	8591	0.012	1	
	12:122702724	rs676770	T>A	NM_177551.3	missense	c.560A>T	p.(Q187L)	0	1296	0	1	8599	0.012	1	
	12:122702730	rs150040336	G>C	NM_177551.3	missense	c.554C>G	p.(T185S)	0	1296	0	2	8598	0.023	1	
	12:122702766	rs144376493	C>G	NM_177551.3	missense	c.517_518delinsAC	p.(G173T)	4	1292	0.309	**0**	**8600**	**0**	2.93e-4	
	12:122702769	rs375831072	C>G	NM_177551.3	missense	c.515G>C	p.(G172A)	0	1296	0	**1**	**8599**	**0.012**	1	
	12:122702781	rs145014727	G>A	NM_177551.3	missense	c.503C>T	p.(P168L)	4	1292	0.309	26	8574	0.302	1	
	12:122702785	rs147573131	T>A	NM_177551.3	missense	c.499A>T	p.(M167L)	4	1292	0.309	28	8572	0.326	1	
	12:122702860	rs151172149	T>C	NM_177551.3	missense	c.424C>T	p.(R142W)	3	1293	0.231	1	8599	0.012	0.002	
	12:122702881	rs140267406	C>T	NM_177551.3	missense	c.403G>A	p.(A135T)	0	1296	0	2	8598	0.023	1	
	12:122702902	rs201756503	G>A	NM_177551.3	missense	c.382C>T	p.(R128W)	0	1296	0	1	8599	0.012	1	
	12:122702911	rs371493470	T>C	NM_177551.3	missense	c.373A>G	p.(R125G)	0	1296	0	1	8599	0.012	1	
	12:122702967	rs144981479	A>C	NM_177551.3	missense	c.317T>G	p.(M106R)	0	1296	0	1	8599	0.012	1	
	12:122703052	rs201537980	T>A	NM_177551.3	missense	c.232A>T	p.(I78F)	0	1296	0	1	8599	0.012	1	
	12:122703081	rs141935401	T>C	NM_177551.3	missense	c.203A>G	p.(N68S)	0	1296	0	4	8596	0.047	1	
	12:122703252	rs755259236	C>T	NM_177551.3	missense	c.32T>C	p.(L11P)	1	1295	0.077	1	8599	0.012	0.131	
	12:122703269	rs147537296	A>C	NM_177551.3	missense	c.15T>G	p.(H5Q)	0	1296	0	2	8598	0.023	1	
	12:122703276	rs140256769	C>T	NM_177551.3	missense	c.8G>A	p.(R3Q)	0	1296	0	1	8599	0.012	1	
HCAR3	12:122715620	unknown	R>A1	NM_006018.2	frameshift	c.1117del1	p.(Q373Kfs*82)	0	1296	0	1	8253	0.012	1	1
	12:122715648	rs377693593	T>C	NM_006018.2	missense	c.1090A>G	p.(N364D)	0	1296	0	1	8599	0.012	1	
	12:122715680	rs200645408	C>T	NM_006018.2	stop-gained	c.1058G>A	p.(W353*)	0	1296	0	1	8599	0.012	1	
	12:122715717	rs371201756	C>T	NM_006018.2	missense	c.1021G>A	p.(A341T)	0	1296	0	2	8590	0.023	1	
	12:122715765	rs201940569	G>A	NM_006018.2	missense	c.973C>T	p.(R325C)	0	1296	0	2	8590	0.023	1	
	12:122715782	rs369401173	C>T	NM_006018.2	missense	c.956G>A	p.(G319D)	0	1296	0	2	8584	0.023	1	
	12:122715986	rs199932037	C>T	NM_006018.2	missense	c.752G>A	p.(R251Q)	0	1296	0	4	8536	0.047	1	
	12:122716023	rs373342696	C>T	NM_006018.2	missense	c.715G>A	p.(V239I)	0	1296	0	1	8579	0.012	1	
	12:122716028	unknown	R>A1	NM_006018.2	coding	c.707_709del3	p.(V236_A237delinsA)	0	1296	0	1	8227	0.012	1	
	12:122716092	rs200839014	G>A	NM_006018.2	missense	c.C646T	p.(R216W)	1	1295	0.077	0	8600	0.000	0.131	
	12:122716164	rs368825608	T>C	NM_006018.2	missense	c.574A>G	p.(M192V)	0	1296	0	1	8599	0.012	1	
	12:122716178	rs373069919	C>T	NM_006018.2	missense	c.560G>A	p.(R187Q)	0	1296	0	1	8599	0.012	1	
	12:122716205	rs478812	A>C	NM_006018.2	missense	c.533T>G	p.(I178S)	0	1296	0	12	8588	0.140	0.614	
	12:122716212	rs200003731	C>A	NM_006018.2	missense	c.526G>T	p.(V176L)	0	1296	0	6	8594	0.070	1	
	12:122716272	rs367957885	C>T	NM_006018.2	missense	c.466G>A	p.(V156I)	0	1296	0	1	8599	0.012	1	
	12:122716292	rs370219885	C>T	NM_006018.2	missense	c.446G>A	p.(C149Y)	0	1296	0	1	8599	0.012	1	
	12:122716314	rs202161035	A>G	NM_006018.2	missense	c.424T>C	p.(W142R)	0	1296	0	3	8597	0.035	1	
	12:122716352	unknown	R>A1	NM_006018.2	frameshift	c.385del1	p.(V129Wfs*8)	0	1296	0	4	8248	0.049	1	

12:122716356	rs371876470	G>A	NM_006018.2	missense	c.382C>T	p.(R128W)	0	1296	0	2	8598	0.023	1
12:122716436	rs201704809	C>T	NM_006018.2	missense	c.302G>A	p.(R101Q)	0	1296	0	3	8597	0.035	1
12:122716465	unknown	R>A1	NM_006018.2	frameshift	c.272del1	p.(S91*fs*1)	0	1296	0	7	8245	0.085	1
12:122716469	rs373921960	C>T	NM_006018.2	missense	c.269G>A	p.(R90H)	0	1296	0	2	8598	0.023	1
12:122716472	rs376873756	C>T	NM_006018.2	missense	c.266G>A	p.(R89Q)	0	1296	0	1	8599	0.012	1
12:122716482	rs3825149	A>T	NM_006018.2	missense	c.256T>A	p.(Y86N)	0	1296	0	2	8598	0.023	1
12:122716658	rs148858491	G>A	NM_006018.2	missense	c.80C>T	p.(A27V)	4	1292	0.309	12	8588	0.140	0.148
12:122716700	rs368127235	A>G	NM_006018.2	missense	c.38T>C	p.(I13T)	0	1296	0	1	8595	0.012	1
12:122716731	rs190370423	G>A	NM_006018.2	missense	c.7C>T	p.(R3W)	1	1295	0.077	1	8599	0.012	0.245
12:122715620	unknown	R>A1	NM_006018.2	frameshift	c.1117del1	p.(Q373Kfs*82)	0	1296	0	1	8253	0.012	1

Figure S1: ***HCAR1*** **mRNA/genomic alignment (NM_032554.3/hg38) and primer locations**: UTR (red), coding (blue), primers (highlighted according to Supplemental Table 1).

```
GCTTTGTGTG CTCTCCAGCA CGGAGTCCGA GGAGGAACAG CGAAGCTGAA  122730733
AATGAAAATT ACTCTGGCGC TGGACCCAAT CGCTCCTCTA CGGCATCAAT  122730683
CTCATCGGAC CCCCCCACCC TACCGCCTCT CAGAAATGAC CACTTTTGCA  122730633
AAATTGCATG CATTTCCAAG CTTCATCCGG CTCCAGGCTT GGCCTCTCCC  122730583
AGAGGCAGGC GGCTTGTGAG ACGGGCTCCA GAGAAAGGAC CTCCCTGGGT  122730533
CTCTCATTTC CTGGCTGAAG TTTCTCTTCT CGCTGCTGTG GCAGCATCCA  122730483
ACCCACACAC ACAGGACCCG CATCCTGGGT GATGAAGTCA GACACGCAGC  122730433
AGCTGGGTGA GTGCTAACGC TCAGATAAGC ATCTGTGCCA TTGTGGGGAC  122730383
TCCCTGGGCT GCTCTGCACC CGGACACTTG CTCTGTCCCC GCCATGTACA  122730333
ACGGGTCGTG CTGCCGCATC GAGGGGGACA CCATCTCCCA GGTGATGCCG  122730283
CCGCTGCTCA TTGTGGCCTT TGTGCTGGGC GCACTAGGCA ATGGGGTCGC  122730233
CCTGTGTGGT TTCTGCTTCC ACATGAAGAC CTGGAAGCCC AGCACTGTTT  122730183
ACCTTTTCAA TTTGGCCGTG GCTGATTTCC TCCTTATGAT CTGCCTGCCT  122730133
TTTCGGACAG ACTATTACCT CAGACGTAGA CACTGGGCTT TTGGGGACAT  122730083
TCCCTGCCGA GTGGGGCTCT TCACGTTGGC CATGAACAGG GCCGGGAGCA  122730033
TCGTGTTCCT TACGGTGGTG GCTGCGGACA GGTATTTCAA AGTGGTCCAC  122729983
CCCCACCACG CGGTGAACAC TATCTCCACC CGGGTGGCGG CTGGCATCGT  122729933
CTGCACCCTG TGGGCCCTGG TCATCCTGGG AACAGTGTAT CTTTTGCTGG  122729883
AGAACCATCT CTGCGTGCAA GAGACGGCCG TCTCCTGTGA GAGCTTCATC  122729833
ATGGAGTCGG CCAATGGCTG GCATGACATC ATGTTCCAGC TGGAGTTCTT  122729783
TATGCCCCTC GGCATCATCT TATTTTGCTC CTTCAAGATT GTTTGGAGCC  122729733
TGAGGCGGAG GCAGCAGCTG GCCAGACAGG CTCGGATGAA GAAGGCGACC  122729683
CGGTTCATCA TGGTGGTGGC AATTGTGTTC ATCACATGCT ACCTGCCCAG  122729633
CGTGTCTGCT AGACTCTATT TCCTCTGGAC GGTGCCCTCG AGTGCCTGCG  122729583
ATCCCTCTGT CCATGGGGCC CTGCACATAA CCCTCAGCTT CACCTACATG  122729533
AACAGCATGC TGGATCCCCT GGTGTATTAT TTTTCAAGCC CCTCCTTTCC  122729483
CAAATTCTAC AACAAGCTCA AAATCTGCAG TCTGAAACCC AAGCAGCCAG  122729433
GACACTCAAA AACACAAAGG CCGGAAGAGA TGCCAATTTC GAACCTCGGT  122729383
CGCAGGAGTT GCATCAGTGT GGCAAATAGT TTCCAAAGCC AGTCTGATGG  122729333
GCAATGGGAT CCCCACATTG TTGAGTGGCA CTGAACAAGC AGACCAACAA  122729283
CACTGAGGAA GATAGAGTGG TGACTTAGAA TTAACTCGTG CTAAGGGGTC  122729233
GGGGGCTTTG AAAATGCCAC CCCCCTTTCT TATTGCAAGA CGGCTTCTCG  122729183
CACATGAACT GCATCCTTCT CATTCTGTCG GAAATGAAAT TCACACAACT  122729133
ATACCTTTTG GGGAGGTTCC AGTTGATTGA AGTGAGTTGG CTGCATTTTC  122729083
TTATCTGATC ACAATGGCAG GGGACAGAAT GTGCATGGAG TGGAGCATGT  122729033
GTGTGTTGGG AGGGGGGCTA GGAACTGCAC AGCCCTTGTG TAATTTTCGT  122728983
TGTTTGTTTT TGTTTTGAGA CAGAGTCTCA CTCTGTGTCC CAGGCTGGAG  122728933
TGCAGTGGCA CAGTCTCGGC TCACTGCAAC CTCTGCCTCC CGGGTTCAAG  122728883
CAATTCTCCT GCCTCAGCCT CCCGAGTAGC TGGGATTAGA GGCGCCAGCC  122728833
AACACACCCG GCTAATTTTT GTATTTTTAG TAGAGACAGG GTTTTGCCAT  122728783
GTTGGCCAGG CTGGTCTCGA GCTCCTGACC TCAGGTGATC CGCCTGCCTT  122728733
GGCCTCCCAA AGTGGTGGGA TCACAGGCGT GAGCCACCGT GCCTGGCCTC  122728683
CCCTGTGTCA TTTTAAATGG CTAAGTAAAT GGGTATATGT GTTTGAATGG  122728633
GGCATGTTCA CTCTCTTAGG GGCTATGGGG CAGTTAGCAG CATTTCCTAT  122728583
CCTCTGACCT TAAATCATTC CTTATCTCAG AAAACAGAAA CCGGGCTCAG  122728533
TCAATCAATG CTTTATTTCA GGCCGAATGA GGCTCTTTAG ATTGGGATCT  122728483
ATTGATCTAT CAATTTTCAT CTTTACATTT CTTTGTACAT CTGTACATTT  122728433
TGTCCAAATG TACATCTGTA CGTCTGTCAT CATTGTGACT TCCTGGTAGC  122728383
CCAAGAAGAA CAACAACAAA ACAATCTGCT CTGACCTTCT TCAAATCTTT  122728333
GTATTTCAAA GAAGGTGCTG AGGGATCTGT TTCCTTGCCC TGGCTTCTCC  122728283
AGTGGGATGT GCTGAGTCCA ATACAATTGC TTTTATAATT GCTTTTGACA  122728233
ACTTGTCATG TGACTGTGAA TTGAAATTAT TCACTTATTT TCCAAGTATT  122728183
TACTGAATTC GTATTTGGTG GCAGGCAGTA TACTGTGTAA TTTTTAGTGG  122728133
AGGGTCATTA GTCAACTCTT ATGTGACAGT AAAGTTTTTT GGGGGGGTGG  122728083
GGACAGAGAA GTTAAGAGCT TTCATCCTTT CACGGAATAC AGTTTCTAGA  122728033
CCGATTCTGT GTGAACATCA GTTTTGTCCT CTTATTGCAA GACTCCCTCA  122727983
TACACATGAG TTTCCCAAAT GTGTACCTGG ACCCCTCGAA ACAGAGGACT  122727933
CTACGAAATG ACAAGCTGCC CCTGCCCTGA ATTAGGGGGA AACATTCCAG  122727883
GCCAACTCTA GCTCCTTTCT CAAGCTACAA AGTGGTGAAC ATGGTTCTCA  122727833
ACTCCTTAAT TTATACTCTC TCAAATGCCC AGGATACTCT ACCCACTTAA  122727783
GAACCTTGCC AACTTCTGGG GGTTGGGCAT GGTGGCTCAC GCTTGTGATC  122727733
CCAGCACTTT GGGAGACTGA GGCGGATCAC CTGAGGTCAG GAGTTCTAGA  122727683
CCAGCCTGAC CAACATGGAG AAACCTCGTC TCTACTAAAA ATTCAAAATT  122727633
AGCCTGGTGT GGTGGCGCAT GCCTATAATC CCAGCTATTC GGGAGGCTGA  122727583
GGCAGGAGAA TCACTTGAAC CGAGGAGGTG GAGGTTGCGG TGAGGTGACA  122727533
TCGTGCCATT GCACTCCAGA CTGGGCCACA GGAGTGAAAC GCCGTCTCAA  122727483
AAAAAAAAAA GAACTTTGCC AACTTCTAGT TCTTTGGCCT CAAAACACCC  122727433
CTCTAGGCTG TCCCTATTAT TCCTTTT
```

Figure S2: *HCAR2* mRNA/genomic alignment (NM_177551.3/hg38) and primer locations: UTR (red), coding (blue), primers (highlighted according to Supplemental Table 2).

```
ATTTAGAAAC CTCCTGCATG GAAGAATTCA TAAGACCAAA ATATTAAAGG  122704988
GAGAAAACAT TCTATAGGAT GACACCCAAT CGCACTGACA AGCAGAGAAA  122704938
CAATGTGTGC AATGGTGTCC CGCTACAAGG CGGGGTTCCA TGTCCTCTTA  122704888
GGTCCTGTCA CTCTAAGTTT AATTTTACAA AAATGGGGGC TTTGAAAATA  122704838
TCACATACTT AGTGGGGGAG GTTTTGCGTG TGTGGAGACA GAGTGCACAG  122704788
GACCGTCCGT ACTTTCTGCT CCATTTTGCT GTGAATCTAA CACTGCCCCA  122704738
AAAAATAAAG TTCATTAAAA AAAATCACGG CCAGGCGCGG TGGCTCACGC  122704688
CTGTAATCCC AGCACTTTGG GAGGCCGAGG CAGACGGATC ACAAGGTCAG  122704638
GAGTTTGAGA CCAGCCTGGC CAACACAGTG AAACCTCGTC TTTACTAAAA  122704588
ATACAAAAAA TTAGCTGGGC GTGGTGGCGC ACGCCTGTAG TCCCAGATAC  122704538
TCAGGAGGCT GAGGCAGAAG AATTGCTTGA ACCTGGGAGG CGGAGGTTGC  122704488
AGTGAGCTGA GATTGTTCCA CCGTTCTCCA GCCTGGGTGA CAGAGTGAGA  122704438
CTCTGTCTCA AAAAAAAAAA ATCACATCCT AAGGTCCCAA GGACATAAAA  122704388
CAAGTTAGAG ACCTCATCCA GTTTTTTGTT TGTTTCAGGG ACCTGCAACC  122704338
AACTTTCTTA CTGACCAGTT TCCTGGGCTG TCTAGAACAG ACAGCTTATG  122704288
GGGTACTAAC CCCATGCTCT ATCCTAAGGT ACCCCTCTTC ATGCAGAGCG  122704238
ACACAGAAAA ACAAATTTGT AGCACAAAGT ACACCCGATT TGCTACAGCT  122704188
TACGACTAGC CTCATGAATC CTTTTTCTCA TTAATTCAAA GTTTGCAAAA  122704138
AAAAGAAATC ACATACTTAA AAATAAATCA CTTGAGCACA GAAGCATATC  122704088
AAAATCTAAT ATAAACAAAC AAAAAACCCA ACTTATGTTA ACATGCCACT  122704038
GAAACTGGGC TCAAGTTTTC AAATGTCTTA TGAAATATTA CACTCTCCAG  122703988
CAGGCTATTT TTTTTTTCCT AACTGGAGAT GAATGGCAGA GTTCTGATAT  122703938
GGAAAAGCAC TCCATGTTTG TACAGAAAGG TAGCATAGGA AAAGTGGTCA  122703888
AATTCAACCA GTCTGTCCTC ATCAAACAGG CAACTACTGT CTTCTCAGCA  122703838
TTTCCAAAAG AAATCATATG TCTGCTACTG TTTATGTCAT ATGCCTGTTA  122703788
ATTTTTCATG CTAATTCTTA GTGTTTCTGG AAAACCTCAC TGTTTGGCAC  122703738
CAAAAATTGT TATTGCTATT GTTGTTGTTT TGTTAATTAT GTCAGCCAAG  122703688
CAGATGACGG TGAATTAATT CTGCAACGTT GAGTACTCAG TTGGCATACA  122703638
AGCACCCAGC CTTCCAAAGG GATGTCCTTC ATGTTTCACT GATTTTCGAA  122703588
TGACAGTCGG CTTGCCTAAA CTTATCCTTT ACATTTAAGA CATTTTGTGG  122703538
GTTCCCGTCT GCCCAGGGGA TTCTTATTAC TTGTTTATGC AAACAGCAGG  122703488
TTGCATAAGA GCCTTGCTGG CTTTTTTTTT TTTTTTTTTT TTTAGAGATT  122703438
TCGTAGTTTC CTGGTAACCA TTCAGTCATC TATTTCAACA CCCTGACATG  122703388
ACATAAAGGC AGGCGTGGAA CCACACGTTC ACCACACAGA CACACACCTC  122703338
CTTGCTGGAG CATTCACTAG GCGAGGCGCT CCATCGGACT CACTAGCCGC  122703288
ACTCATGAAT CGGCACCATC TGCAGGATCA CTTTCTGGAA ATAGACAAGA  122703238
AGAACTGCTG TGTGTTCCGA GATGACTTCA TTGTCAAGGT GTTGCCGCCG  122703188
GTGTTGGGGC TGGAGTTTAT CTTCGGGCTT CTGGGCAATG GCCTTGCCCT  122703138
GTGGATTTTC TGTTTCCACC TCAAGTCCTG GAAATCCAGC CGGATTTTCC  122703088
TGTTCAACCT GGCAGTGGCT GACTTTCTAC TGATCATCTG CCTGCCCTTC  122703038
CTGATGGACA ACTATGTGAG GCGTTGGGAC TGGAAGTTTG GGGACATCCC  122702988
TTGCCGGCTG ATGCTCTTCA TGTTGGCTAT GAACCGCCAG GGCAGCATCA  122702938
TCTTCCTCAC GGTGGTGGCG GTAGACAGGT ATTTCCGGGT GGTCCATCCC  122702888
CACCACGCCC TGAACAAGAT CTCCAATCGG ACAGCAGCCA TCATCTCTTG  122702838
CCTTCTGTGG GGCATCACTA TTGGCCTGAC AGTCCACCTC CTGAAGAAGA  122702788
AGATGCCGAT CCAGAATGGC GGTGCAAATT TGTGCAGCAG CTTCAGCATC  122702738
TGCCATACCT TCCAGTGGCA CGAAGCCATG TTCCTCCTGG AGTTCTTCCT  122702688
GCCCCTGGGC ATCATCCTGT TCTGCTCAGC CAGAATTATC TGGAGCCTGC  122702638
GGCAGAGACA AATGGACCGG CATGCCAAGA TCAAGAGAGC CATCACCTTC  122702588
ATCATGGTGG TGGCCATCGT CTTTGTCATC TGCTTCCTTC CCAGCGTGGT  122702538
TGTGCGGATC CGCATCTTCT GGCTCCTGCA CACTTCGGGC ACGCAGAATT  122702488
GTGAAGTGTA CCGCTCGGTG GACCTGGCGT TCTTTATCAC TCTCAGCTTC  122702438
ACCTACATGA ACAGCATGCT GGACCCCGTG GTGTACTACT TCTCCAGCCC  122702388
ATCCTTTCCC AACTTCTTCT CCACTTTGAT CAACCGCTGC CTCCAGAGGA  122702338
AGATGACAGG TGAGCCAGAT AATAACCGCA GCACGAGCGT CGAGCTCACA  122702288
GGGGACCCCA ACAAAACCAG AGGCGCTCCA GAGGCGTTAA TGGCCAACTC  122702238
CGGTGAGCCA TGGAGCCCCT CTTATCTGGG CCCAACCTCT CCTTAAATAA  122702188
CCATGCCAAG AAGGGACATT GTCACCAAGA ACCAGGATCT CTGGAGAAAC  122702138
AGTTGGGCTG TTGCATCGAG TAATGTCACT GGACTCGGCC TAAGGTTTCC  122702088
TGGAACTTCC AGATTCAGAG AATGCGATTT AGGGAAACGG TGGCAGATGA  122702038
GTGGGAGACT GGTTGCAAGG TGTGACCGCA GGAATCCTGG AGGAATAGAG  122701988
AGTAAAGCTT CTAGGCATCT GAAACTTTTG CTTCATCTCT GACGCTCGCA  122701938
GGACTGAAGA TGGGCAAATT GTAGGCATTT CTGCTGAGCA GAGTTGGAGC  122701888
CAGAGATCTA CTTGTGACTT GTTGGCCTTC TTCCCACATC TGCCTCAGAC  122701838
TGGAGGGGGC TCAGCTCCTG GGGTGATATC TAGCCTGCTT GTGAGCTCTA  122701788
GCAGGGATAA GGAGAGCTGA GATTGGAGGG AATTGTGTTG CTCCTGGAGG  122701738
GAGCCCAGGC ATCATTAAAC AAGCCAGTAG GTCACCTGGC TTCCGTGGAC  122701688
CAATTCATCT TTCAGACAAG CTTTAGCAGA AATGGACTCA GGGAAGAGAC  122701638
TCACACGCTT TGGTTAATAT CTGTGTTTCC GGTGGGTGTA ATAGGGGATT  122701588
AGCCCCAGAA GGGACTGAGC TAAACAGTGT TATTATGGGA AAGGAAATGG  122701538
```

```
CATTGCTGCT TTCAACCAGC GACTAATGCA ATCCATTCCT CTCTTGTTTA  122701488
TAGTAATCTA AGGGTTGGGC AGTTAAAACG GCTTCAGGAT AGAAAGCTGT  122701438
TTCCCACCTC TGTTTGCTTT TAACATTAAA AGGGAAATGT GCCTCTGCCC  122701388
CACAGTTAGA GGGGTGCACG TTCCTCCTGG TTCCTTCGCT TGTGTTTCTG  122701338
TACTTACCAA AAATCTACCA TTTCAATAAA TTTTGATAGG AGACAGTCTG  122701288
GCGTTGCTAT ATCTGGGTTA TGGTTCACAG AAATGGACTT CTTACTTAAG  122701238
CAGAGACCTT TATGGGTTGT TAACTCTGTC CTGGTAGAAA ACTACTCCAC  122701188
ACTGCAAAGT AAGAAAGAGA CACAGACACT GAGCTACATC ATGAAACAAC  122701138
TAAGCAGAAA TTACACCTGT GTCTAAACAA AGATAGAAAG AGCTGGGATT  122701088
AAAAATAATT TGGAAGGATA GTACAGATAG GATCGCAAGA GCACTTACAT  122701038
TTTATTAGAG TTTGGTGAAT ATTGCTCTTA GTATTTGTGC ATTAAACAGG  122700988
GAATCAAGGC CAGGTGCAAT GGTTCATGCC TGTAATCCCA GCACTTTGAG  122700938
GGGACCGAGG TGGGCAGATC ACCCTGAGGT CAGGAGTTTG AGACCAGCCT  122700888
GGACAACATG GCGAAACCCC ACTTCTACTA AAAATACAAG AAAAATTAGC  122700838
CAGGCATGGT GGCGGCCGCT TGTAGTCCCA GCTACTGGGA AGGCTGGGGG  122700788
AAGAAGAATT GCTTGAACCC AGGAGGCGGA GGTTGCAGTG AGCCGAGGTG  122700738
GTGTGCCTGC ACTCCAGCCT GGGGGACAGA GCAAGACTTC ATCTCAAATA  122700688
ATAATAATAA ATAATATAAA AAGGGAATCA AGCCAGGCAG ATATTTTTCC  122700638
ATAGGAGGGG GAAGAGCGAG CTTCCTATGG AATGGTTTCA GTTCCCAATT  122700588
CAGAGAAGGA AAGGAGATGT ACTGGTTTGA GTCTGTGATC AGTCCAAGTT  122700538
TCTGTTACGC TTTCATTTCT AACTCTGTCA GTCCTTGATT ATTTGGCAAG  122700488
ACGAGGCTTG CTTCAACCTC CTGACATGAA CACAGATAAG GAATTTAGAG  122700438
ACAGGCTCCC AGCTCTGGGT GAATGACAGT AAATGAAGAA AGGTGGATGC  122700388
TCTGGAATTA CCTCTGTGCT TTCAGAGCAG TTGGCTTGTC TGTATCACCA  122700338
AGCAATGTAC AGGTAAAAAG CCAAAAGCAA GGATCACCTT AAAGAGGAAG  122700288
AGATGTCAGG GATGAGCTCA GAGGAAAATA GGGATTCCCC AGCCTCGGAG  122700238
```

Figure S3: *HCAR3* mRNA/genomic alignment (NM_006018.2/hg38) and primer locations: UTR (red), coding (blue), primers (highlighted according to Supplemental Table 3).

```
CCACTGAAAC TGGGCTCAAG TTTTCAAACA TCTTATGAAA TATTACACTC  122717443
TCCAGCAGGC TTTTTTTTTT TTCCTAACTG GAGATGAATG GCAGAGTTCT  122717393
GATATGGAAA AGCACTCCAT GTTTGTACAG AAAGGTAGCA TAGGAAAAGT  122717343
GGTCAAATTC AACCAGTCTG TCCTCATCAA ACAGGCAACT ACTGTCTTCT  122717293
CAGCATTTCC AAAAGAAATC ATATGCCTGC TACTGTTTAT GTCATATGCC  122717243
TGTTAATTTT TCATGCTAAT TCTTAGTGTT TCTGGAAAAC CTCACTGTTT  122717193
GGCACCAAAA ATTATTATTG CTATTGTTGT TGTTTTGTTA ATTATGTCAG  122717143
CCAAGCAGAT GACGGTGAAT TAATTCTGCA ACGTTGAGTA CTCAGTTGGC  122717093
ATACAAGCAC CCAGCCTTCC AAAGGGATGT CCTTCATGTT TCACTGATTT  122717043
TCGAATGACA GTCAGCTTGC CTAAACTTAT CCTTTACATT TAAGACATTT  122716993
GGTGGGTTCC TGTATGCCCA GGGGATTTTT ATTATTTGTT TATGCAAACA  122716943
GCAGGTTGCA TAAGAGCCTT GCTGGCTTTT TTTTTTTTTT TTTTAGAGAT  122716893
TTCGTAGTTT CCTGATAACC ATTCAGTCAT CTATTTCAAC ACCCTGACAT  122716843
GACATAAAGG CAGGCACGGA ACCACACGTT CACCATACAG ACACACGCCA  122716793
CTTTGCTGGA GCATTCACTA GGCGAGGCGC TCCATCGGAC TCACTAGCTG  122716743
CACTCATGAA TCGGCACCAT CTGCAGGATC ACTTTCTGGA AATAGACAAG  122716693
AAGAACTGCT GTGTGTTCCG AGATGACTTC ATTGCCAAGG TGTTGCCGCC  122716643
GGTGTTGGGG CTGGAGTTTA TCTTTGGGCT TCTGGGCAAT GGCCTTGCCC  122716593
TGTGGATTTT CTGTTTCCAC CTCAAGTCCT GGAAATCCAG CCGGATTTTC  122716543
CTGTTCAACC TGGCAGTAGC TGACTTTCTA CTGATCATCT GCCTGCCGTT  122716493
CGTGATGGAC TACTATGTGC GGCGTTCAGA CTGGAAGTTT GGGGACATCC  122716443
CTTGCCGGCT GGTGCTCTTC ATGTTTGCCA TGAACCGCCA GGGCAGCATC  122716393
ATATTCCTCA CGGTGGTGGC GGTAGACAGG TATTTCCGGG TGGTCCATCC  122716343
CCACCACGCC CTGAACAAGA TCTCCAATTG GACAGCAGCC ATCATCTCTT  122716293
GCCTTCTGTG GGGCATCACT GTTGGCCTAA CAGTCCACCT CCTGAAGAAG  122716243
AAGTTGCTGA TCCAGAATGG CACTGCAAAT GTGTGCATCA GCTTCAGCAT  122716193
CTGCCATACC TTCCGGTGGC ACGAAGCTAT GTTCCTCCTG GAGTTCTTCC  122716143
TGCCCCTGGG CATCATCCTG TTCTGCTCAG CCAGAATTAT CTGGAGCCTG  122716093
CGGCAGAGAC AAATGGACCG GCATGCCAAG ATCAAGAGAG CCATCACCTT  122716043
CATCATGGTG GTGGCCATCG TCTTTGTCAT CTGCTTCCTT CCCAGCGTGG  122715993
TTGTGCGGAT CCACATCTTC TGGCTCCTGC ACACTTCGGG CACGCAGAAT  122715943
TGTGAAGTGT ACCGCTCGGT GGACCTGGCG TTCTTTATCA CTCTCAGCTT  122715893
CACCTACATG AACAGCATGC TGGACCCCGT GGTGTACTAC TTCTCCAGCC  122715843
CATCCTTTCC CAACTTCTTC TCCACTTTGA TCAACCGCTG CCTCCAGAGG  122715793
AAGATAACAG GTGAGCCAGA TAATAACCGC AGCACGAGCG TCGAGCTCAC  122715743
AGGGGACCCC AACAAAACCA GAGGCGCTCC AGAGGCGTTA ATCGCCAACT  122715693
CCGGTGAGCC ATGGAGCCCC TCTTATCTGG GCCCAACCTC AAATAACCAT  122715643
TCCAAGAAGG GACATTGTCA CCAAGAACCA GCATCTCTGG AGAAACAGTT  122715593
GGGCTGTTGC ATCGAGTAAT GTCACTGGAC TCGGCCTAAG ATTTCCTGGA  122715543
ACTTCCAGAT TCAGAGAATC TGATTTAGGG AAACTGTGGC AGATGAGTGG  122715493
GAGACTGGTT GCAAGGTGTG ACCGCAGGAA TCCTGGAGGA ACAGAGAGTA  122715443
AAGCTTCTAG GCATCTGAAA CTTGCTTCAT CTCTGACGCT CGCAGGACTG  122715393
AAGATGGGCA AATTGTAGGC GTTTCTGCTG AGCAGAGTTG GAGCCAGAGA  122715343
TCTACTTGTG ACTTGTTGGC CTTCTTCCCA CATCTGCCTC AGACTGGAGG  122715293
GGGCTCAGCT CCTGGGGTGA TATCTAGCCT GCTTGTGAGC TCTAGCAGGG  122715243
ATAAGGAGAG CTGAGATTGG AGGGAATTGT GTTGTTCCTG GAGGAAGCCC  122715193
AGGCATCATT AAACAAGCCA GTAGGTCACC TGGCTTCCGT GGACCAATTC  122715143
ATCTTTCAGA CAATCTTTAG CAGAAATGGA CTCAGGGAAG AGACTCACAT  122715093
GCTTTGGTTA GTATCTGTGT TTCCGGTGGG TGTAATAGGG GATTAGCCCC  122715043
AGAAGGGACT GAGCTAAACA GTGTTATTAT GGGAAAGGAA ATGGCATTGC  122714993
TGCTTTCAAC CAGCGACTAA TGCAATCCAT TCCTCTCTTG TTTATAGTAA  122714943
TCTAAGGGTT GAACAGTTAA AACGGCTTCA GGATAGAAAG CTGTTTCCCA  122714893
CCTGTTTGCT TTTACCATTA AAAGGGAAAC GTGCCTCTGC CCCACGGGTA  122714843
GAGGGGTGCA CGTTCCTCCT GGTTCCTTCG CTTGTGTTTC TGTACTTACC  122714793
AAAAATCTAC CATTTCAATA AATTTTGATA GGAGACAGTC TGGCGTTGCT  122714743
ATATCTGGGT TATGGTTCAC AGAAATGGAC TTCTTACTTA AGCAGAGACC  122714693
TTTATGGGTT GTTAACTTTG TCCTGGTAGA AAACTACTCC ACACTGCAAA  122714643
GTAAGAAAGA GACACAGACA CTGAGCTACA TCATGAAACA ACTAAGCAGA  122714593
AATTACACCT GTGTCTAAAC AAAGATAGAA AGAGCTGGGA TTAAAAATAA  122714543
TTTGGAAGGA TAGTACAGAT AGGATTGCAA GAGCACTTAC ATTTTATTAG  122714493
AGTTTGGTGA ATATTGCTCT TCATATTTGT GCATTAAAAA GGGAATCAAG  122714443
GCCAGGTGCA ATGGTTCATG CCTGTAATCC CAGCACTTTG GGGGAGCCAA  122714393
GGCGGGCAGA TCACCTGAAG TAAGGAGTTT GAGACCAGCC TGGACAAAAT  122714343
GGTGAAACCC CACTTCTACT AAAAATACAA GAAAAATTAG CCAGGCATGG  122714293
TGGCGGCCGC TTGTAGTCCC AGCTACTGGG AAGGCTGGGA GAAGAAGAAT  122714243
TGCTTGAACC CGGGAGGCGG AGGTTGCAGT GAGCCGAGGT GGTGTGCCTG  122714193
CACTCCAGCC TGGGGGACAG AGCAAGACTT CATCTCA
```

Figure S4: Assessment of *HCAR3* internal primer set C.

- **Panel A**: UCSC In-silico PCR of internal primer set C generated two PCR products of 600bp, one targeting *HCAR3* and the other *HCAR2*.
- **Panel B**: Blastn compared the two amplicons from panel A; with 98% identity, a total of 13 differences were identified. The positions highlighted grey or red were selected to visualize in electropherograms in panels C and D.
- **Panel C**: Electropherogram segments of internal primer set C nested PCR. Arrows highlight positions from panel B that varied between the two amplicons and demonstrate that only *HCAR3* was amplified. These three segments were from the BC-affected individual from the AHCC who has *HCAR3* c.560G>A (p.R187Q), which is marked red (amplicon position 185).
- **Panel D**: Electropherogram segment of internal primer set C nested PCR. This is a segment from another BC-affected individual from the AHCC who did not have *HCAR3* c.560G>A (p.R187Q) (amplicon position 185). *HCAR3* c.560G>A (p.R187Q) was only detected in 1 out of the 46 BC-affected individuals screened in this study from the AHCC. This segment also demonstrates that only the *HCAR3* allele was amplified at amplicon position 198.

A

```
UCSC In-Silico PCR
Forward primer: TATTTCCGGGTGGTCCATCC
Reverse primer: GCGGTTATTATCTGGCTCACC

>HCAR3:chr12:122715763-122716362 600bp
TATTTCCGGGTGGTCCATCCccaccacgccctgaacaagatctccaattg
gacagcagccatcatctcttgccttctgtggggcatcactgttggcctaa
cagtccacctcctgaagaagaagttgctgatccagaatggcactgcaaat
gtgtgcatcagcttcagcatctgccataccttccggtggcacgaagctat
gttcctcctggagttcttcctgcccctgggcatcatcctgttctgctcag
ccagaattatctggagcctgcggcagagacaaatggaccggcatgccaag
atcaagagagccatcaccttcatcatggtggtggccatcgtctttgtcat
ctgcttccttcccagcgtggttgtgcggatccacatcttctggctcctgc
acacttcgggcacgcagaattgtgaagtgtaccgctcggtggacctggcg
ttctttatcactctcagcttcacctacatgaacagcatgctggaccccgt
ggtgtactacttctccagcccatcctttcccaacttcttctccactttga
tcaaccgctgcctccagaggaagataacaGGTGAGCCAGATAATAACCGC

>HCAR2:chr12:122702309-122702908 600bp
TATTTCCGGGTGGTCCATCCccaccacgccctgaacaagatctccaatcg
gacagcagccatcatctcttgccttctgtggggcatcactattggcctga
cagtccacctcctgaagaagaagatgccgatccagaatggcggtgcaaat
ttgtgcagcagcttcagcatctgccataccttccagtggcacgaagccat
gttcctcctggagttcttcctgcccctgggcatcatcctgttctgctcag
ccagaattatctggagcctgcggcagagacaaatggaccggcatgccaag
atcaagagagccatcaccttcatcatggtggtggccatcgtctttgtcat
ctgcttccttcccagcgtggttgtgcggatccgcatcttctggctcctgc
acacttcgggcacgcagaattgtgaagtgtaccgctcggtggacctggcg
ttctttatcactctcagcttcacctacatgaacagcatgctggaccccgt
ggtgtactacttctccagcccatcctttcccaacttcttctccactttga
tcaaccgctgcctccagaggaagatgacaGGTGAGCCAGATAATAACCGC
```

B

```
Identities:587/600(98%),  Gaps:0/600(0%), Strand: Plus/Plus

HCAR3  1    TATTTCCGGGTGGTCCATCCCCACCACGCCCTGAACAAGATCTCCAATTGGACAGCAGCC  60
            |||||||||||||||||||||||||||||||||||||||||||||| |||||||||||||
HCAR2  1    TATTTCCGGGTGGTCCATCCCCACCACGCCCTGAACAAGATCTCCAATCGGACAGCAGCC  60

HCAR3  61   ATCATCTCTTGCCTTCTGTGGGGCATCACTGTTGGCCTAACAGTCCACCTCCTGAAGAAG  120
            |||||||||||||||||||||||||||||| ||||||| |||||||||||||||||||||
HCAR2  61   ATCATCTCTTGCCTTCTGTGGGGCATCACTATTGGCCTGACAGTCCACCTCCTGAAGAAG  120

HCAR3  121  AAGTTGCTGATCCAGAATGGCACTGCAAATGTGTGCATCAGCTTCAGCATCTGCCATACC  180
            ||| ||| |||||||||||||  ||||||| |||||| ||||||||||||||||||||||
HCAR2  121  AAGATGCCGATCCAGAATGGCGGTGCAAATTTGTGCAGCAGCTTCAGCATCTGCCATACC  180

HCAR3  181  TTCCGGTGGCACGAAGCTATGTTCCTCCTGGAGTTCTTCCTGCCCCTGGGCATCATCCTG  240
            |||| |||||||||| ||||||||||||||||||||||||||||||||||||||||||||
HCAR2  181  TTCCAGTGGCACGAAGCCATGTTCCTCCTGGAGTTCTTCCTGCCCCTGGGCATCATCCTG  240

HCAR3  241  TTCTGCTCAGCCAGAATTATCTGGAGCCTGCGGCAGAGACAAATGGACCGGCATGCCAAG  300
            ||||||||||||||||||||||||||||||||||||||||||||||||||||||||||||
HCAR2  241  TTCTGCTCAGCCAGAATTATCTGGAGCCTGCGGCAGAGACAAATGGACCGGCATGCCAAG  300

HCAR3  301  ATCAAGAGAGCCATCACCTTCATCATGGTGGTGGCCATCGTCTTTGTCATCTGCTTCCTT  360
            ||||||||||||||||||||||||||||||||||||||||||||||||||||||||||||
HCAR2  301  ATCAAGAGAGCCATCACCTTCATCATGGTGGTGGCCATCGTCTTTGTCATCTGCTTCCTT  360

HCAR3  361  CCCAGCGTGGTTGTGCGGATCCACATCTTCTGGCTCCTGCACACTTCGGGCACGCAGAAT  420
            |||||||||||||||||||||| |||||||||||||||||||||||||||||||||||||
HCAR2  361  CCCAGCGTGGTTGTGCGGATCCGCATCTTCTGGCTCCTGCACACTTCGGGCACGCAGAAT  420

HCAR3  421  TGTGAAGTGTACCGCTCGGTGGACCTGGCGTTCTTTATCACTCTCAGCTTCACCTACATG  480
            ||||||||||||||||||||||||||||||||||||||||||||||||||||||||||||
HCAR2  421  TGTGAAGTGTACCGCTCGGTGGACCTGGCGTTCTTTATCACTCTCAGCTTCACCTACATG  480

HCAR3  481  AACAGCATGCTGGACCCCGTGGTGTACTACTTCTCCAGCCCATCCTTTCCCAACTTCTTC  540
            ||||||||||||||||||||||||||||||||||||||||||||||||||||||||||||
HCAR2  481  AACAGCATGCTGGACCCCGTGGTGTACTACTTCTCCAGCCCATCCTTTCCCAACTTCTTC  540

HCAR3  541  TCCACTTTGATCAACCGCTGCCTCCAGAGGAAGATAACAGGTGAGCCAGATAATAACCGC  600
            ||||||||||||||||||||||||||||||||||| ||||||||||||||||||||||||
HCAR2  541  TCCACTTTGATCAACCGCTGCCTCCAGAGGAAGATGACAGGTGAGCCAGATAATAACCGC  600
```

C

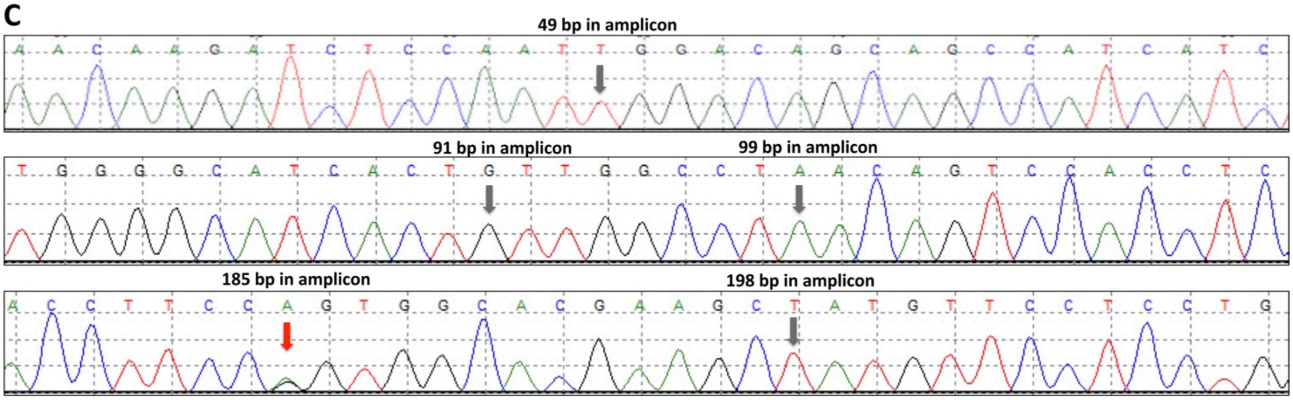

D

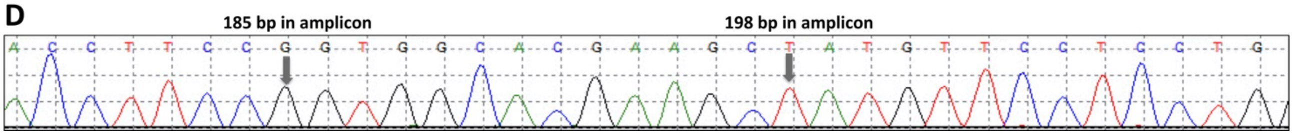

Figure S5: IGV view of *HCAR2* variants detected in TCGA, highlighting each variant and HCAR2/3 differences.

Alignment is reverse complement. Yellow is mutation location. Red is HCAR2/3 difference demonstrating that HCAR2 was sequenced.

HCAR2: n.106T>C; c.32T>C; p.L11P

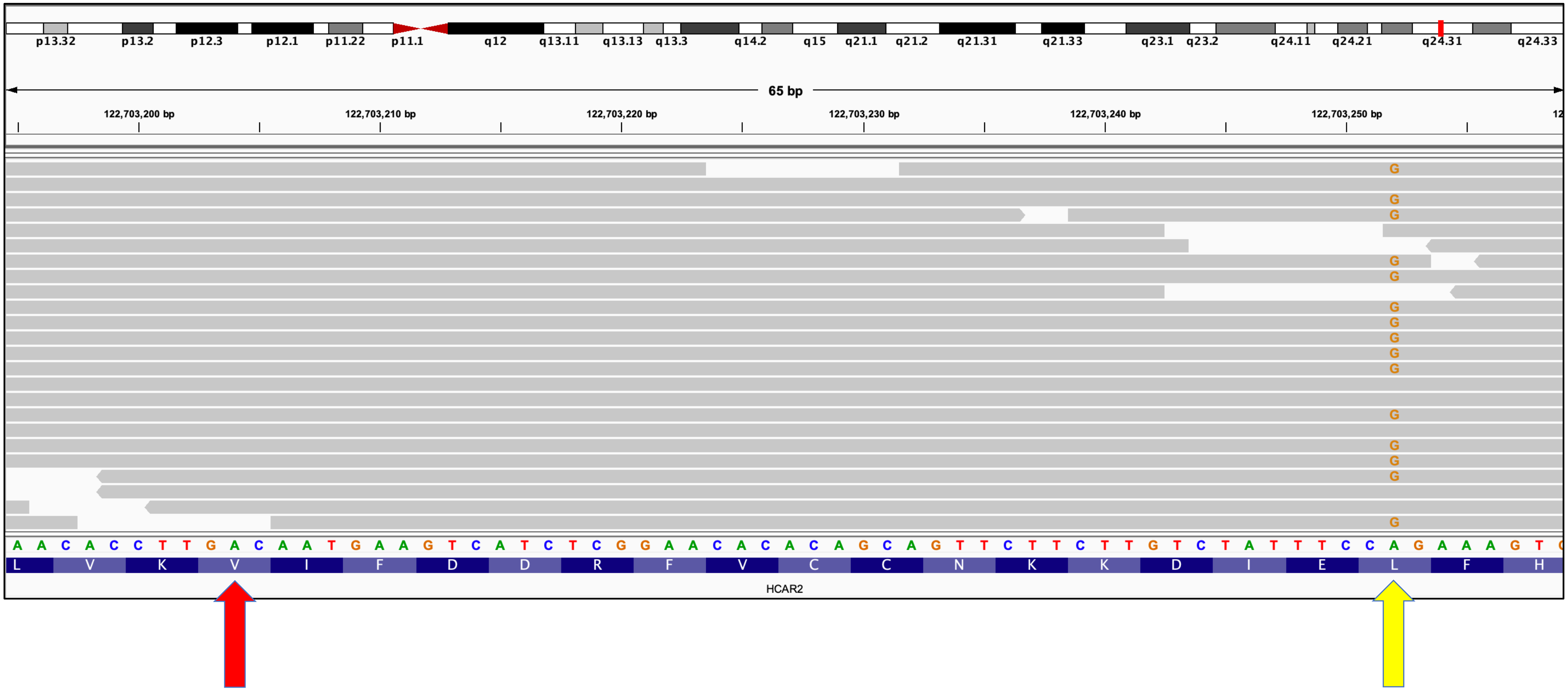

HCAR2 CACTAGCCGCACTCATGAATCGGCACCATCTGCAGGATCACTTTCTGGAAATAGACAAGA n.120

HCAR3 CACTAGCTGCACTCATGAATCGGCACCATCTGCAGGATCACTTTCTGGAAATAGACAAGA n.120

HCAR2 AGAACTGCTGTGTGTTCCGAGATGACTTCATTGTCAAGGTGTTGCCGCCGGTGTTGGGGC n.180

HCAR3 AGAACTGCTGTGTGTTCCGAGATGACTTCATTGCCAAGGTGTTGCCGCCGGTGTTGGGGC n.180

HCAR2: n.498C>T; c.424C>T; p.R142W

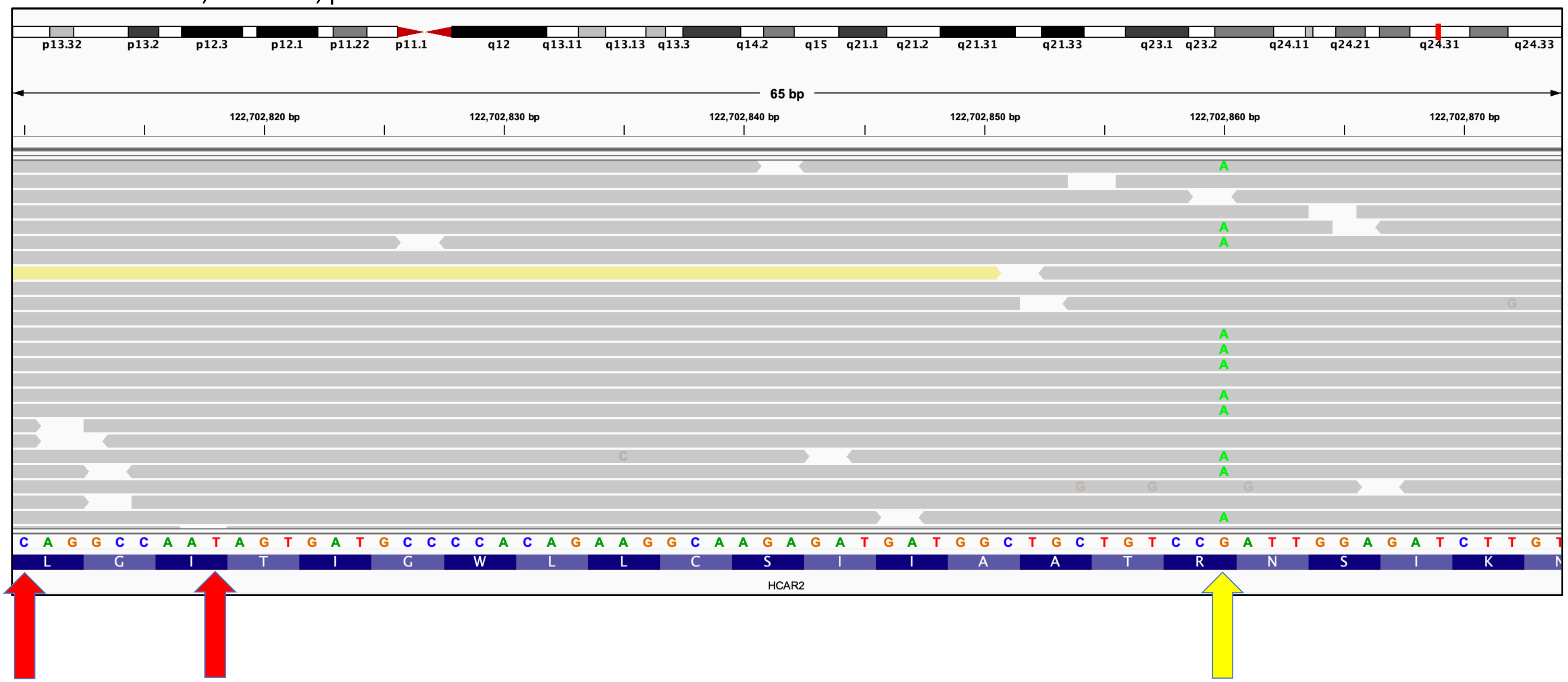


```
HCAR2   TGAACAAGATCTCCAATCGGACAGCAGCCATCATCTCTTGCCTTCTGTGGGGCATCACTA   n.540
        ||||||||||||||||| ||||||||||||||||||||||||||||||||||||||||| 
HCAR3   TGAACAAGATCTCCAATTGGACAGCAGCCATCATCTCTTGCCTTCTGTGGGGCATCACTG   n.540

HCAR2   TTGGCCTGACAGTCCACCTCCTGAAGAAGAAGATGCCGATCCAGAATGGCGGTGCAAATT   n.600
        ||||||| ||||||||||||||||||||||| ||| |||||||||||||  |||||||| 
HCAR3   TTGGCCTAACAGTCCACCTCCTGAAGAAGAAGTTGCTGATCCAGAATGGCACTGCAAATG   n.600
```

HCAR2: n.591_592delinsAC; c.517_518delinsAC; p.G173T

HCAR2: n.577C>T; c.503C>T; p.P168L

HCAR2: n.573A>T; c.499A>T; p.M167L

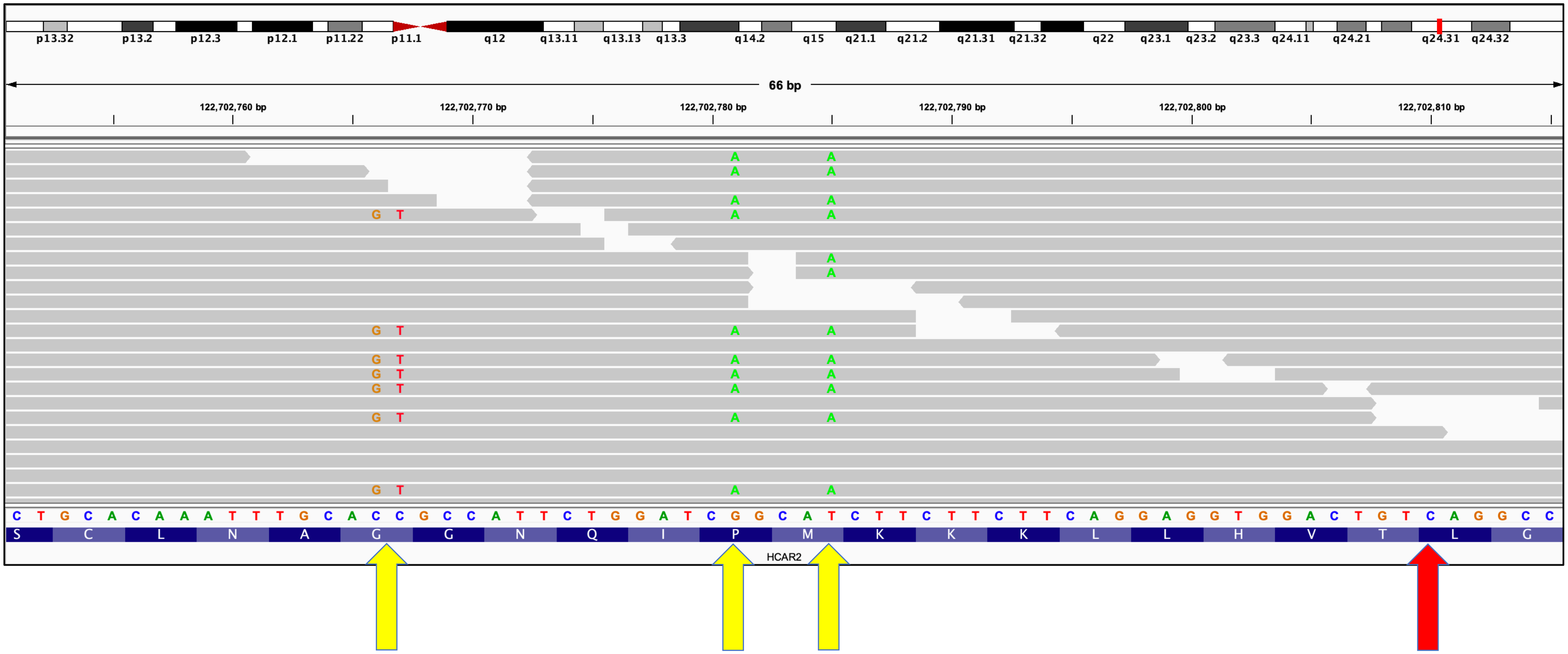


```
HCAR2  TTGGCCTGACAGTCCACCTCCTGAAGAAGAAGATGCCGATCCAGAATGGCGGTGCAAATT  n.600
       ||||||| ||||||||||||||||||||||||| ||| |||||||||||||  ||||||||
HCAR3  TTGGCCTAACAGTCCACCTCCTGAAGAAGAAGTTGCTGATCCAGAATGGCACTGCAAATG  n.600
```

HCAR2: n.1006G>A; c.932G>A; p.R311H

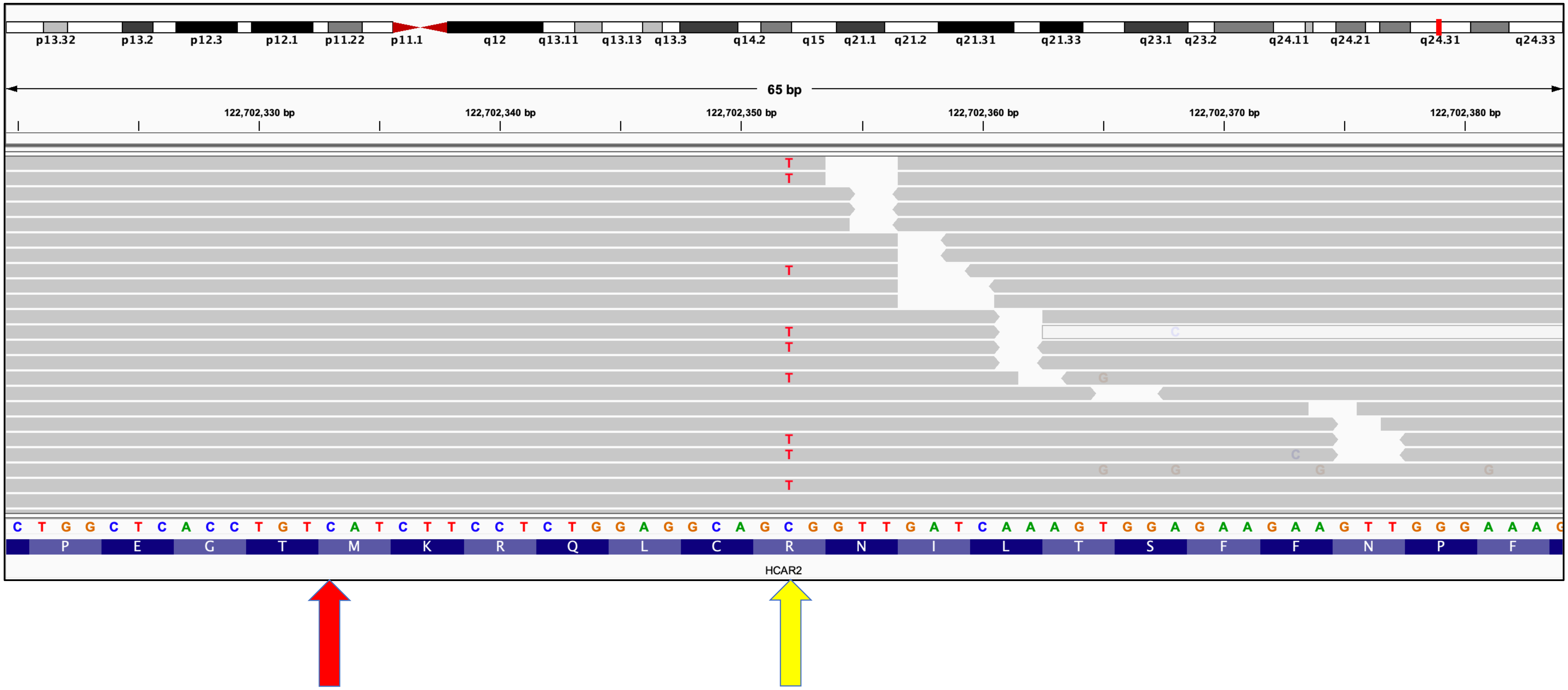


```
HCAR2  TCTCCAGCCCATCCTTTCCCAACTTCTTCTCCACTTTGATCAACCGCTGCCTCCAGAGGA  n.1020
       ||||||||||||||||||||||||||||||||||||||||||||||||||||||||||||
HCAR3  TCTCCAGCCCATCCTTTCCCAACTTCTTCTCCACTTTGATCAACCGCTGCCTCCAGAGGA  n.1020

HCAR2  AGATGACAGGTGAGCCAGATAATAACCGCAGCACGAGCGTCGAGCTCACAGGGGACCCCA  n.1080
       |||| |||||||||||||||||||||||||||||||||||||||||||||||||||||||
HCAR3  AGATAACAGGTGAGCCAGATAATAACCGCAGCACGAGCGTCGAGCTCACAGGGGACCCCA  n.1080
```

HCAR2: n.1110A>G; c.1036A>G; p.M346V

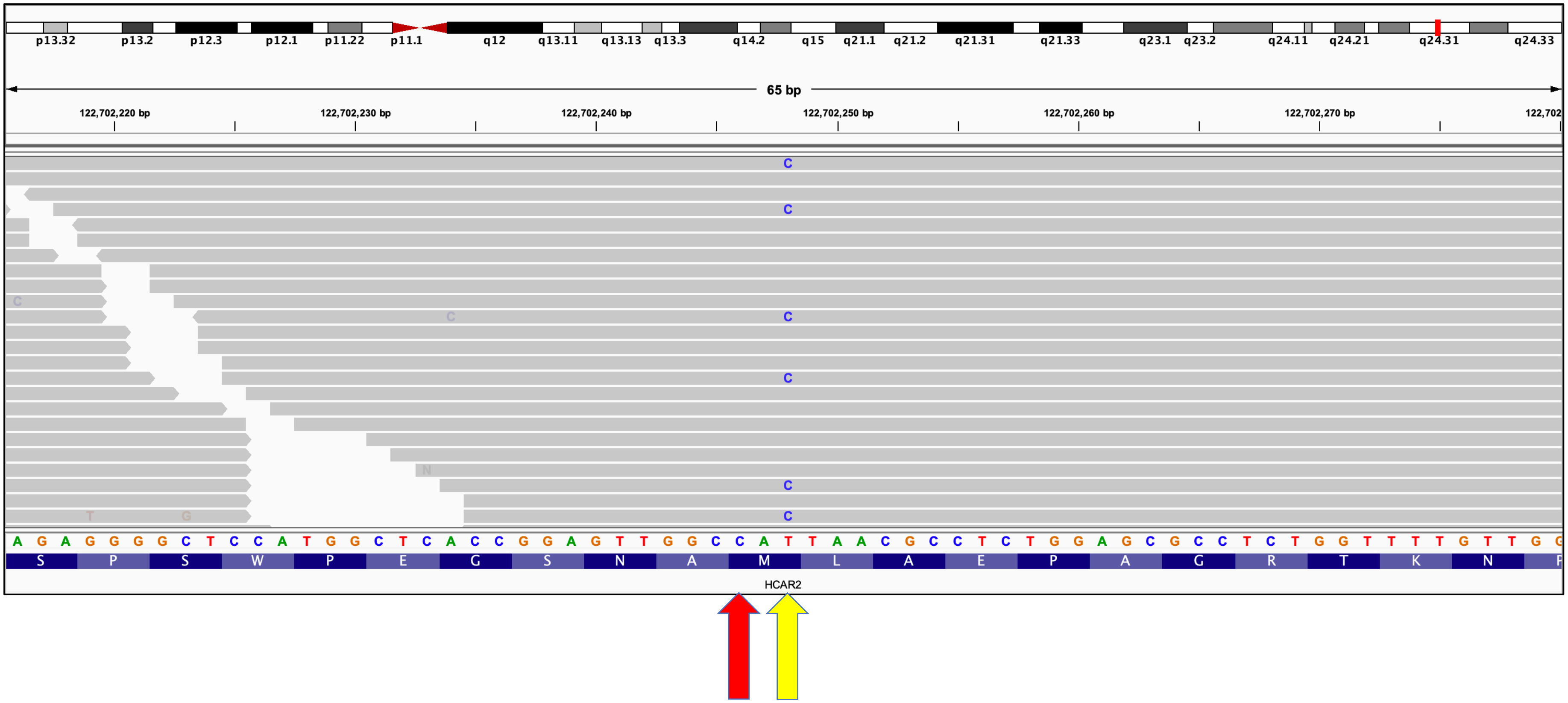


```
HCAR2  ACAAAACCAGAGGCGCTCCAGAGGCGTTAATGGCCAACTCCGGTGAGCCATGGAGCCCCT  n.1140
       ||||||||||||||||||||||||||||||| |||||||||||||||||||||||||||||
HCAR3  ACAAAACCAGAGGCGCTCCAGAGGCGTTAATCGCCAACTCCGGTGAGCCATGGAGCCCCT  n.1140
```

HCAR2: n.1122G>A; c.1048G>A; p.G350S

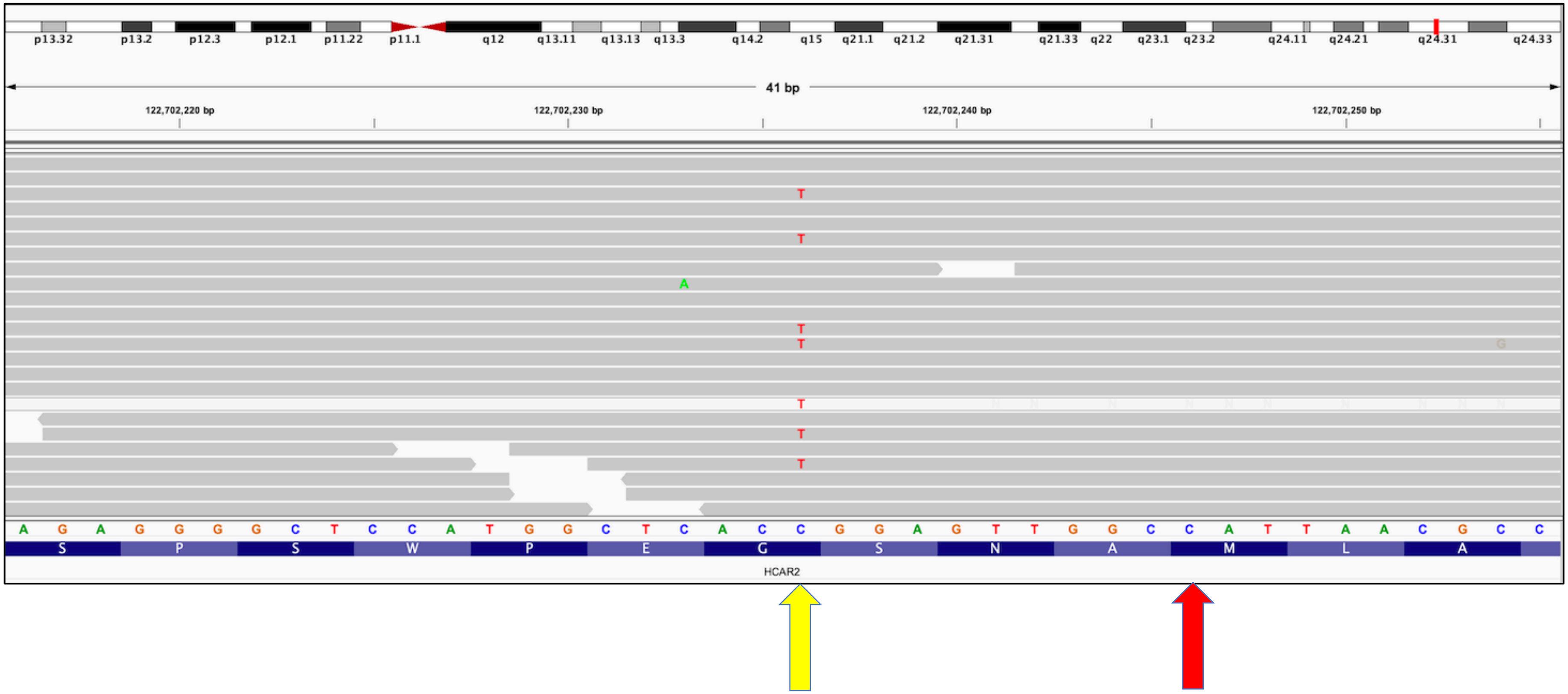


```
HCAR2  ACAAAACCAGAGGCGCTCCAGAGGCGTTAATGGCCAACTCCGGTGAGCCATGGAGCCCCT  n.1140
       ||||||||||||||||||||||||||||||| ||||||||||||||||||||||||||||
HCAR3  ACAAAACCAGAGGCGCTCCAGAGGCGTTAATCGCCAACTCCGGTGAGCCATGGAGCCCCT  n.1140
```

HCAR2: n.1160_1164del; c.1086_1090del; p.P363Nfs*26

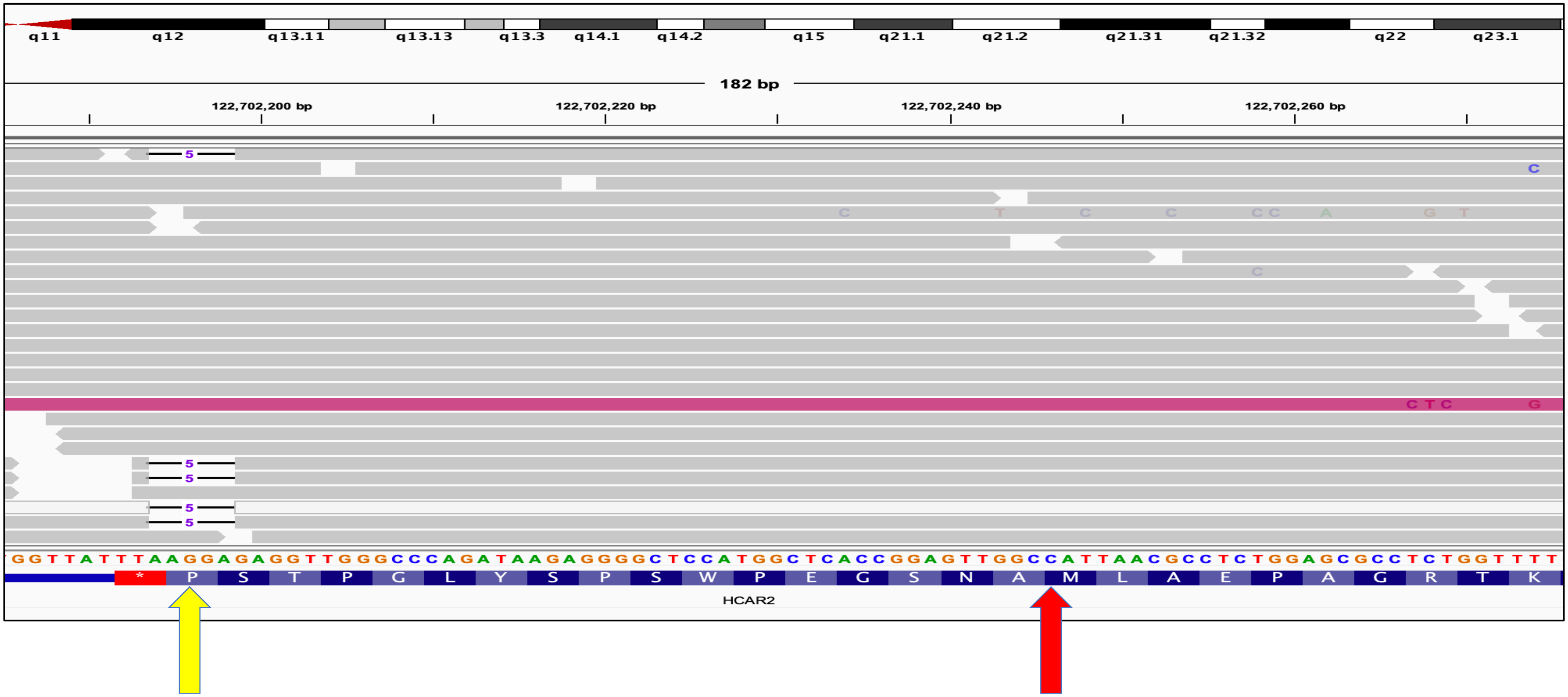


```
HCAR2  ACAAAACCAGAGGCGCTCCAGAGGCGTTAATGGCCAACTCCGGTGAGCCATGGAGCCCCT  n.1140
       ||||||||||||||||||||||||||||||| ||||||||||||||||||||||||||||
HCAR3  ACAAAACCAGAGGCGCTCCAGAGGCGTTAATCGCCAACTCCGGTGAGCCATGGAGCCCCT  n.1140

HCAR2  CTTATCTGGGCCCAACCTCTCCTTAAATAACCATGCCAAGAAGGGACATTGTCACCAAGA  n.1200
       |||||||||||||||||||     |||||||||| |||||||||||||||||||||||||
HCAR3  CTTATCTGGGCCCAACCTC-----AAATAACCATTCCAAGAAGGGACATTGTCACCAAGA  n.1195
```

Figure S6: IGV view of *HCAR3* variants detected in TCGA, highlighting each variant and HCAR2/3 differences.

Alignment is reverse complement. Yellow is mutation location. Red is HCAR2/3 difference demonstrating that HCAR3 was sequenced.

HCAR3: n.154C>T; c.80C>T; p.A27V

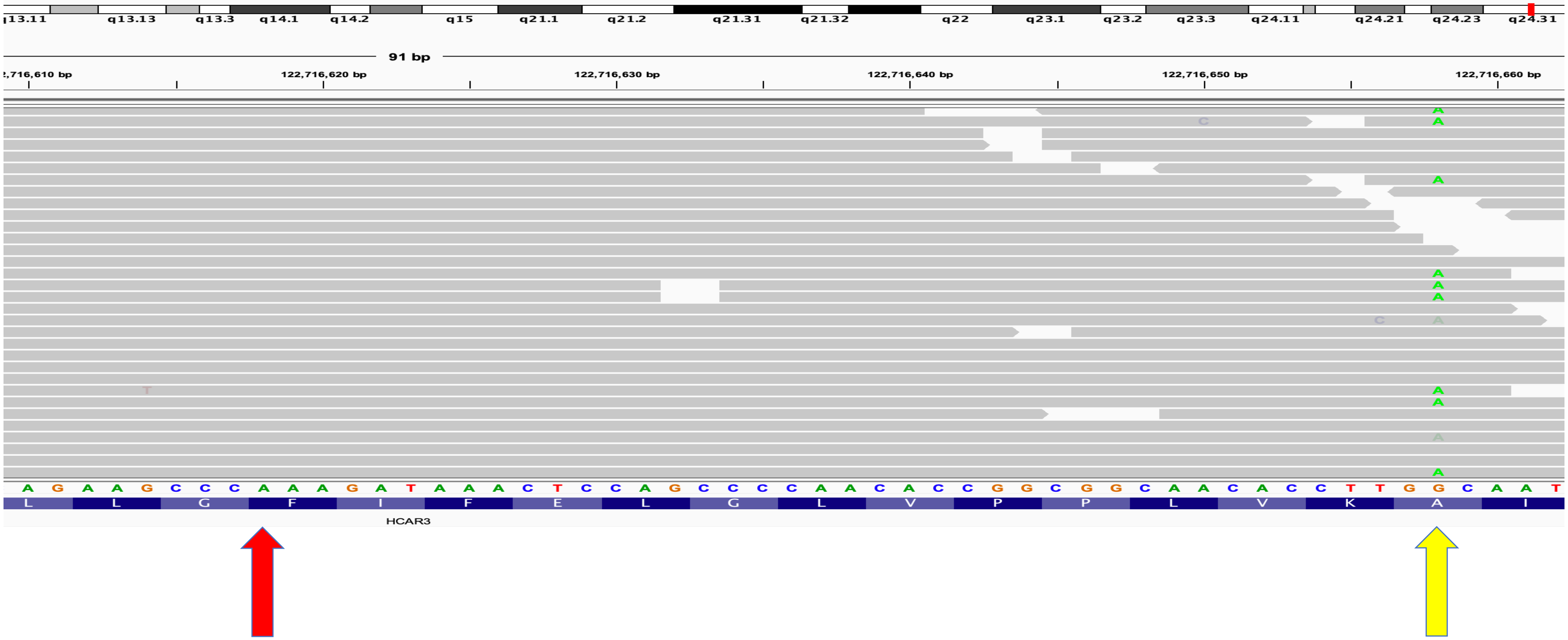


```
HCAR2  AGAACTGCTGTGTGTTCCGAGATGACTTCATTGTCAAGGTGTTGCCGCCGGTGTTGGGGC  n.180
       ||||||||||||||||||||||||||||||||| ||||||||||||||||||||||||||
HCAR3  AGAACTGCTGTGTGTTCCGAGATGACTTCATTGCCAAGGTGTTGCCGCCGGTGTTGGGGC  n.180

HCAR2  TGGAGTTTATCTTCGGGCTTCTGGGCAATGGCCTTGCCCTGTGGATTTTCTGTTTCCACC  n.240
       ||||||||||||| ||||||||||||||||||||||||||||||||||||||||||||||
HCAR3  TGGAGTTTATCTTTGGGCTTCTGGGCAATGGCCTTGCCCTGTGGATTTTCTGTTTCCACC  n.240
```

HCAR3: n.81C>T; c.7C>T; p.R3W

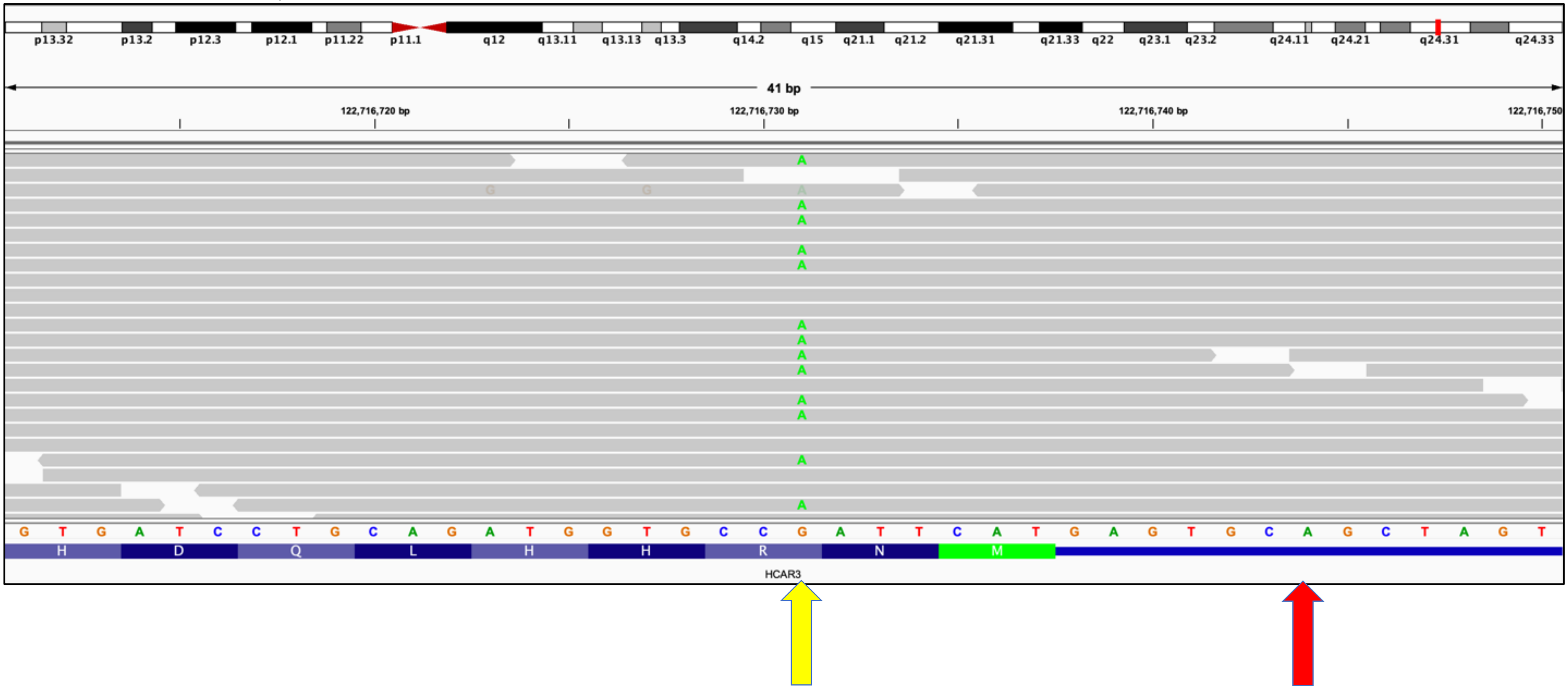


```
HCAR2  CACTAGCCGCACTCATGAATCGGCACCATCTGCAGGATCACTTTCTGGAAATAGACAAGA  n.120
       ||||||| ||||||||||||||||||||||||||||||||||||||||||||||||||||
HCAR3  CACTAGCTGCACTCATGAATCGGCACCATCTGCAGGATCACTTTCTGGAAATAGACAAGA  n.120
```

Figure S7: IGV view of *HCAR2/3* variants detected in TCGA not reported because original call was based on misalignment.

Alignment is reverse complement. Yellow is mutation location. Red is HCAR2/3 difference.

HCAR3: n.1122G>A; c.1048G>A; p.G350S

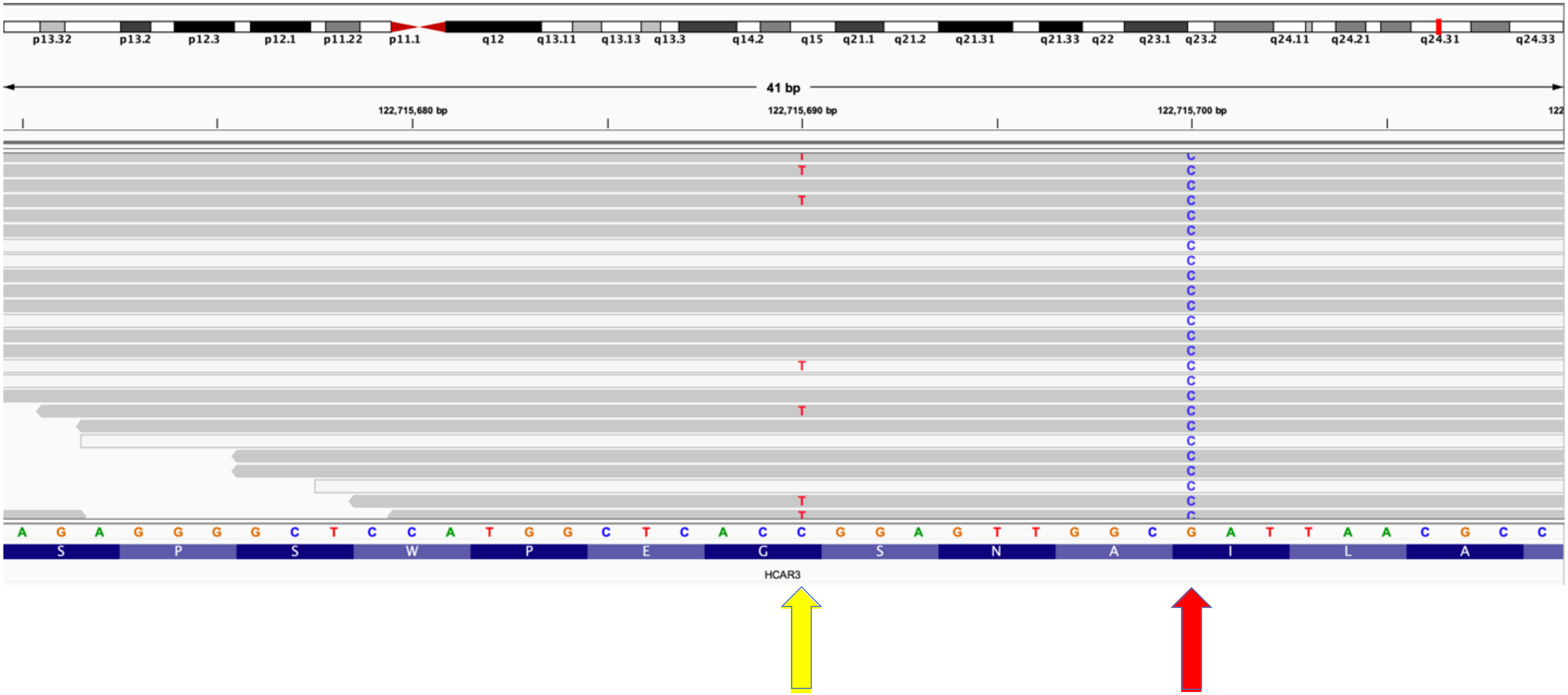


```
HCAR2   ACAAAACCAGAGGCGCTCCAGAGGCGTTAATGGCCAACTCCGGTGAGCCATGGAGCCCCT   n.1140
        ||||||||||||||||||||||||||||||| ||||||||||||||||||||||||||||
HCAR3   ACAAAACCAGAGGCGCTCCAGAGGCGTTAATCGCCAACTCCGGTGAGCCATGGAGCCCCT   n.1140
```

HCAR3: n.1110A>G; c.1036A>G; p.I346V

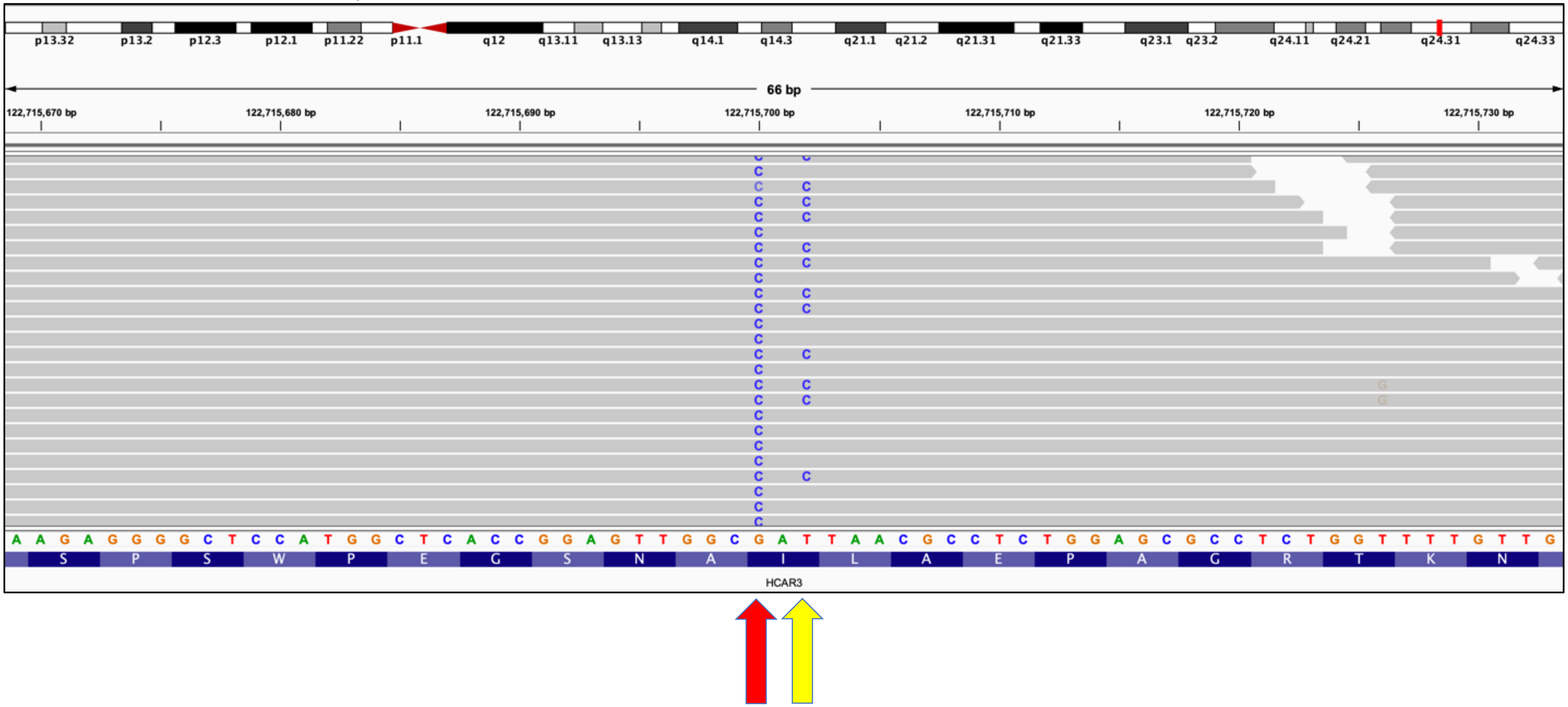


```
HCAR2  ACAAAACCAGAGGCGCTCCAGAGGCGTTAATGGCCAACTCCGGTGAGCCATGGAGCCCCT  n.1140
       ||||||||||||||||||||||||||||||| ||||||||||||||||||||||||||||
HCAR3  ACAAAACCAGAGGCGCTCCAGAGGCGTTAATCGCCAACTCCGGTGAGCCATGGAGCCCCT  n.1140
```

HCAR2: n.666T>C; c.592T>C; p.F198L

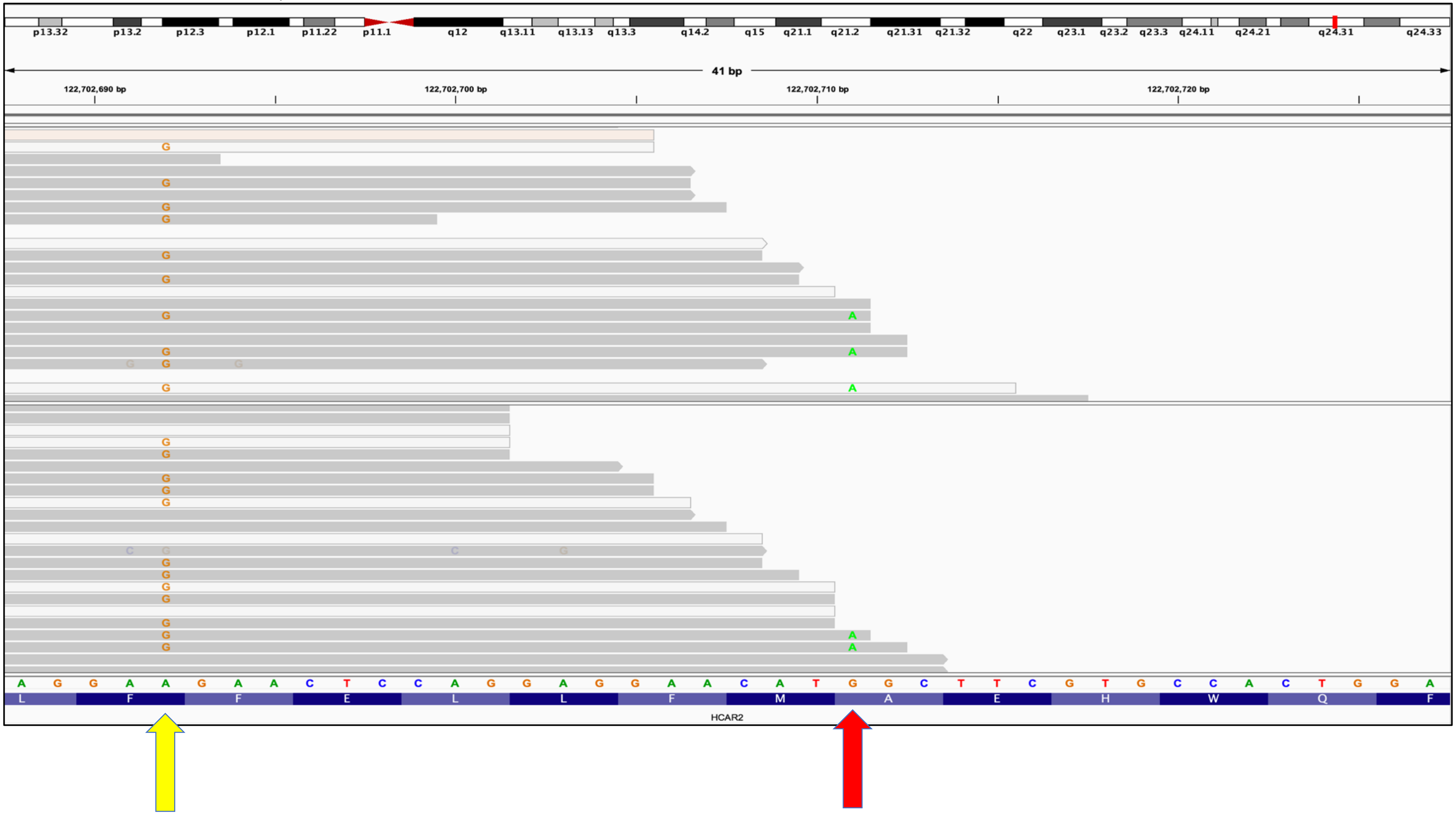


```
HCAR2  TGTGCAGCAGCTTCAGCATCTGCCATACCTTCCAGTGGCACGAAGCCATGTTCCTCCTGG n.660
       |||||| |||||||||||||||||||||||||| |||||||||||| |||||||||||||
HCAR3  TGTGCATCAGCTTCAGCATCTGCCATACCTTCCGGTGGCACGAAGCTATGTTCCTCCTGG n.660

HCAR2  AGTTCTTCCTGCCCCTGGGCATCATCCTGTTCTGCTCAGCCAGAATTATCTGGAGCCTGC n.720
       ||||||||||||||||||||||||||||||||||||||||||||||||||||||||||||
HCAR3  AGTTCTTCCTGCCCCTGGGCATCATCCTGTTCTGCTCAGCCAGAATTATCTGGAGCCTGC n.720
```
